# Fine tuning the morphology of peptide amphiphile nanostructures *via* co-assembly†

**DOI:** 10.1039/d5sc02935j

**Published:** 2025-07-03

**Authors:** Maria Mercedes Fiora, Huihua Xing, Marilina Cathcarth, Octavio Garate, Santiago Herrera, Agustin S. Picco, Gabriel Ybarra, Martin Conda-Sheridan, Mario Tagliazucchi

**Affiliations:** a Instituto Nacional de Tecnología Industrial, Micro y Nanotecnologías San Martín B1650WAB Buenos Aires Argentina; b Universidad de Buenos Aires, Facultad de Ciencias Exactas y Naturales, Departamento de Química Inorgánica Analítica y Química Física Pabellón 2, Ciudad Universitaria, Ciudad Autónoma de Buenos Aires C1428 Argentina mario@qi.fcen.uba.ar; c Department of Pharmaceutical Sciences, College of Pharmacy, University of Nebraska Medical Center Omaha NE 68198 USA martin.condasheridan@unmc.edu; d Instituto de Investigaciones Fisicoquímicas, Teóricas y Aplicadas (INIFTA), UNLP, CONICET Diagonal 113 y Calle 64 1900 La Plata Argentina; e Universidad de Buenos Aires, Consejo Nacional de Investigaciones Cientificas y Tecnicas, Facultad de Ciencias Exactas y Naturales, Instituto de Quimica de los Materiales, Ambiente y Energía Pabellón 2, Ciudad Universitaria, Ciudad Autonoma de Buenos Aires C1428 Argentina

## Abstract

The self-assembly of peptide amphiphiles (PAs) in aqueous solution yields nanoconstructs displaying a rich spectrum of sizes and morphologies, including micelles, fibers, and lamellar ribbons. The morphology impacts the bioactivity of the PAs and, thus, efforts have been made to control it by tuning their molecular structure or the solution pH. However, synthesizing new PAs is time consuming and biomedical applications limit the pH to physiologically relevant ranges. This work demonstrates that the composition of a binary mixture of co-assembled PAs serves as a powerful approach to exert rational control over the morphology, size and transition pHs of the supramolecular nanostructures. We combined light scattering, SAXS, TEM and AFM experiments and theoretical predictions using a Molecular Theory (MOLT) to construct composition–pH morphology diagrams for three relevant PA mixtures. For C_16_KK/C_16_KKK mixtures (C_16_: palmitoyl and K: lysine), we demonstrate fine tuning of the micelle-to-fiber transition pH by varying the composition of the system. For a mixture of oppositely charged PAs, C_16_EEE/C_16_KKK (E: glutamic acid), theory and experiments reveal interesting composition-driven micelle-to-fiber-to-micelle transitions. The C_16_KK/C_16_EE mixture exhibits three different morphologies—micelles, fibers, and lamellae—and regions of the morphology diagram showing coexistence between fibers and lamellae. MOLT calculations also provide insights into the internal organization of the assemblies and predict that the nanostructure radius can also be tuned by the composition of the mixture, in agreement with SAXS observations.

## Abstract 2

The composition of a mixture of co-assembled peptide amphiphiles is a simple yet powerful parameter to exert control over the size and morphology of their self-assembled nanoconstructs.

## Graphical abstract

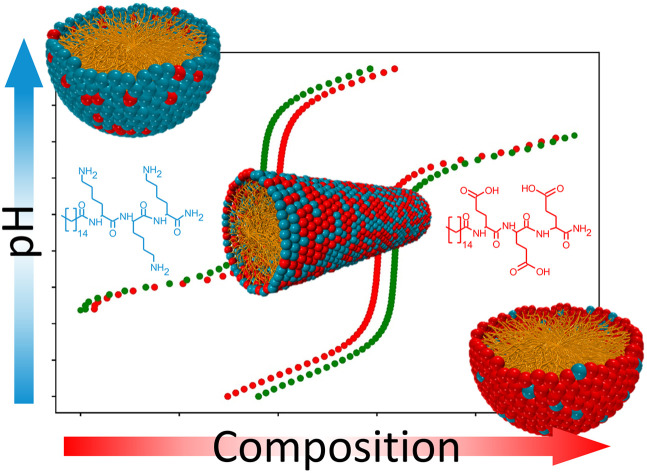

## 1 Introduction

Peptide amphiphiles (also known as lipopeptides) are a family of self-assembling molecules that combine a peptidic headgroup and an hydrophobic tail, usually comprising a linear alkyl chain.^1–4^ These molecules exhibit a rich self-assembling behavior in solution, forming a variety of nanostructures that include spherical micelles,^5–8^ short (∼100 nm)^9,10^ and long (>10 μm)^7,8,10–12^ cylindrical fibers and planar ribbons.^5,6,12^ The inclusion of bioactive epitopes in the peptidic headgroup (such as IKVAV) imparts biological activity to the assemblies. Notably, PA's bioactivity depends on the morphology of the nanostructures,^10,12–14^ for example Stupp's group studied a series of PAs terminated in the IKVAV sequence, which assembled into different morphologies.^12^ PAs forming non-aggregated cylindrical fibers displayed the IKVAV sequence more efficiently than those assembling into bundles of ribbons, and, therefore, showed higher bioactivity for neurite outgrowth.^12^ In another example from the same group, the length and aggregation state of PA nanofibers determined their activity in promoting cell adhesion and survival.^10^ This effect was attributed to the fact that long fibers displayed higher adhesive forces than short ones, which benefited cell spreading and proliferation. In a third example, Conda-Sheridan and co-workers studied the antimicrobial activity of a family cationic PAs and showed that PAs assembling into micelles were more active than those forming fibers or ribbons.^13^ This behavior was ascribed to the weak cohesivity of spherical micelles compared to fibers, which favored their disassembly into isolated PAs that disrupted bacterial membranes.

The relevance of morphology to bioactivity calls for approaches to exert control over the shape, size, charge and mesoscale aggregation of these self-assembled nanostructures. So far, the most important variables used to manipulate these properties have been the molecular architecture of the PA, the solution pH,^5–8,15^ the presence and type of counterions,^16,17^ and the ionic strength.^18^ For example, the PAs of the family C_n_K_m_ (where C_n_ and K_m_ indicate n methyl/methylene groups and m lysine units, respectively) display spherical micelle → cylindrical fiber → lamellar ribbon transitions with increasing solution pH.^18–21^ These transitions have been quantitatively predicted using a theoretical tool known as Molecular Theory (MOLT),^20,21^ and can be rationalized in terms of the deprotonation of the side-chain amino groups in the lysines with increasing pH. Lower protonation degrees result in weaker electrostatic repulsions among headgroups, which decreases the curvature of the nanostructure. There are also some general design rules to control morphology by introducing structural modifications in the PA molecule. Introducing cohesive β-sheet-forming amino acids near the hydrophobic core is known to favor the formation of fibers over spherical micelles because of the formation of hydrogen bonds aligned with the long axis of the fiber.^22,23^ On the other hand, decreasing the length of the alkyl tail^18,24^ or increasing the size of the peptidic headgroup,^7,20^ tends to favor small micelles over nanofibers or ribbons, in qualitative agreement with Israelachvili's packing theory.^25^ These examples demonstrate morphology control through molecular architecture or solution composition, but there are limits to the practical usefulness of these approaches. Changing molecular architecture is time-consuming and cannot be done gradually, which is a disadvantage for fine-tuning the aggregate properties. On the other hand, biomedical applications restrict the solution pH and ionic strength to physiologically relevant values. In this work, we explore PA co-assembly as a novel strategy to finely control the morphology and size of the nanostructures.

The co-assembly of PAs has been previously addressed by different groups,^11,15,26–34^ with special emphasis in the combination of bioactive PAs with shorter PAs that act as diluents or fillers,^26–28^ and mixtures of positively and negatively charged small PAs.^15,29–32,35^ In an interesting example of the latter, Wester *et al.*^35^ showed that equimolar mixtures of C_16_KKK/C_16_EEE and C_16_(K)_5_K/C_16_(E)_5_E (E: glutamic acid) tend to form nanofibers, even when one or both individual components form spherical micelles. This is consistent with the behavior of other mixtures of positively/negatively charged PAs^15,29–32^ and it has been ascribed to the increase in cohesiveness due to electrostatic interactions between the oppositely charged headgroups. However, most of these studies were restricted to a few PA compositions and solution pH values, which precluded the construction of systematic morphology diagrams. On the other hand, the co-assembly of like-charged small PAs has been much less investigated than that of oppositely charged ones. In the present work, we comprehensively examine the morphology behavior of mixtures of like- and oppositely charged PAs. For this purpose, we use a molecular theory (MOLT) that we previously developed for single-component PA nanostructures^20^ and apply it here for the first time to PA mixtures. We performed Light Scattering (LS), Atomic Force Microscopy (AFM), Transmission Electron Microscopy (TEM), and Small Angle X-ray Scattering (SAXS) experiments for selected systems and demonstrate that MOLT predictions are in good agreement with these experimental observations. In mixtures of like-charged PAs (C_16_KKK/C_16_KK) our results reveal that co-assembly allows manipulation of the micelle → fiber transition pH by varying the composition of the mixture. We identify regions of morphology coexistence, where micelles and fibers coincide over a narrow composition range. In mixtures of oppositely charged PAs like C_16_KKK/C_16_EEE, we observed a composition-driven micelle → fiber → micelle transition, governed by electrostatic interactions and headgroup protonation states. Additionally, we observed a complex morphology behavior in C_16_KK/C_16_EE mixtures, with transitions between micelles, fibers, and lamellae depending on composition and pH, highlighting the interplay between headgroup charge and tail cohesive interactions. SAXS analysis provided insights into structural parameters, such as core radius and shell thickness and showed an increase in the micelle radius in C_16_KKK/C_16_EEE as the mixture composition approaches charge stoichiometry, in agreement with MOLT calculations.

## 2 Methods

### 2.1 Experimental methods

#### 2.1.1 PA synthesis

The synthesis of the PAs C_16_KK, C_16_KKK, C_16_EE and C_16_EEE was performed using standard Fmoc solid-phase peptide synthesis, as described previously.^20^ Amino-acids and rink amide resins were supplied by P3Bio and solvents were supplied by Fisher. All amino-acids used in this study were L-enantiomers. PA structures were confirmed by MALDI and ^1^H NMR (see the ESI†).

#### 2.1.2 LS

Light scattering measurements were performed using a Dynamic Light Scattering instrument (Wyatt DynaPro NanoStar) equipped with a 658 nm laser. The scattered light was collected at a detection angle of 90°. All solutions were prepared at a concentration of 0.25% w/v in borate buffer. For each composition, the pH was adjusted by adding small amounts of HCl or NaOH as needed. After pH adjustment, samples were allowed to age for one day and were measured the following day.

#### 2.1.3 TEM

TEM experiments were performed on a FEI Tecnai G2 Spirit for 1 : 1 (molar fraction of 0.5) mixtures of C_16_KK and C_16_KKK peptides at different pH values and a total PA concentration of 0.25% w/v. The pH of the solution was adjusted using buffer solutions.

#### 2.1.4 AFM

A small volume of the PA solution (0.25% w/v) was deposited onto a mica substrate, allowing adsorption for 1 minute, and the excess solution was gently removed and dried with nitrogen to ensure uniform coverage. AFM imaging was performed in air using an Agilent 5500 scanning probe microscope (Agilent Technologies) isolated from vibrations, air turbulence and acoustic noise. Images were acquired using an insulating triangular Si tip (PointProbes Plus Non-Contact/Soft Tapping Mode, radius > 10 nm, force constant 48 N m^−1^, resonance frequency 309.1 kHz) and analyzed and edited using Gwyddion®.

#### 2.1.5 SAXS

SAXS measurements were performed at the Cateretê Beamline of the Brazilian Synchrotron Light Laboratory (LNLS-Sirius, Campinas, Brazil). Measurements were performed using an X-ray wavelength of 1.5498 Å and a sample-to-detector distance of 1.75 m. Scattering data were collected using a PIMEGA 540D detector. Samples for SAXS were prepared at 0.25% w/v concentration and their pH values were adjusted using borate buffer. Quartz capillaries (outer diameter of 1.5 mm and wall thickness 0.01 mm) were used to contain the samples and placed in a sample-holder in a vacuum. The accessible scattering vector range under these conditions was *q* = 0.009–0.28 Å^−1^. All measurements were conducted at 25 °C. Scattering data were modelled using SasView 5.0 (https://www.sasview.org/).

### 2.2 Theoretical methods

#### 2.2.1 Molecular theory for amphiphile self-assembly

We studied the mixtures of PAs using a thermodynamical statistical tool known as MOLT. In the past, MOLT has been applied to a broad spectra of problems related to the self-assembly of soft materials.^36^ We have recently applied this tool to model single-component PAs,^20,21^ and extend the tool here to mixtures of PAs.

We provide here an outline of MOLT, and refer the interested reader to the ESI† for a detailed derivation. MOLT is formulated starting from a free energy functional of the system, Ω*(*T*, *V*, *N*_PA_, {*μ*_*i*_}) (the symbol * indicates that the aggregate is fixed in space^25^). This functional describes a system containing fixed numbers *n*_1_ and *n*_2_ of the two PA components in the mixture, PA_1_ and PA_2_, at fixed volume *V* and temperature *T*. The mobile ions in the system (salt anions and cations, H^+^ and OH^−^) have constant chemical potentials {*μ*_*i*_}, which are fixed by the pH and ionic strength of the bulk solution. It should be noted that given this definition, Ω* is a semigrand canonical free energy (canonical for the PAs and grand canonical for the small ions). As explained in detail in our previous studies,^20,36,37^Ω* is written as the combination of different contributions, such as the translational and conformational entropy of the PAs, the energies from short-range effective interactions, the electrostatic interactions and the free energies associated to the acid–base chemical equilibrium. Note that these contributions depend on explicit PA conformations and, therefore, MOLT takes into consideration the chemical structure of the molecules (at a coarse grain level similar to that of coarse-grained MD simulations^26,38^). The contributions to Ω* depend on functions that are unknown *a priori*, such as the local densities of each species, the probability distribution functions of the PA conformations, the local electrostatic potential and the position-dependent degree of ionization of the acid–base groups in the system. Finding the functional extrema of Ω* with respect to these unknown functions results in a system of coupled integro-differential equations, which we solve using numerical methods. This procedure yields both structural information (morphology, size and charge of the aggregates and internal organization) and thermodynamic parameters (such as the free energy per PA, *ω*) for the system in equilibrium. For more information about the formulation of MOLT for amphiphiles, we refer the reader to the ESI† and our previous studies.^20,36,37^

We finally want to mention some characteristics and limitations of the theory. MOLT is a coarse-grained approach, in which groups of ∼4 non-H atoms in the molecular structure are clumped together in a coarse-grained bead (similarly to the MARTINI MD force-field^39^). Therefore, while MOLT incorporates some molecular information (coarse-grained molecular structure and conformations) at a lower cost than MD simulations, it does not provide structural details at the atomistic level and cannot describe some effects that strongly depend on them, such as the effect of amino-acid chirality.^34,40,41^ MOLT predictions are also dependent on a proper parametrization of the bead–bead interactions, which is discussed in the ESI.†

#### 2.2.2 Determination of the equilibrium morphology of PA mixtures and morphology coexistence

To use MOLT to predict the equilibrium morphology for PA mixtures, we solve the theory for different ideal morphologies: spherical micelles, infinitely long cylindrical nanofibers and planar lamellae. These calculations require fixing the experimental conditions of the solution (pH and ionic strength) and the molecular structure and molar fractions of PA_1_ and PA_2_ in the mixture. For each morphology, we first scan the aggregation number, *n*_T_ = *n*_1_ + *n*_2_, and find the one that minimizes the free energy per PA molecule, *ω*.^20,37^ We stress that the calculation is performed for a fixed PA composition, given by the molar fraction *x*_1_ = *n*_1_/*n*_T_. While for single-PA assemblies, the structure with the lowest *ω* is predicted to be the equilibrium one, the analysis is more complicated in mixtures of PAs due to the possibility of morphology coexistence, as we explain next.

## 3 Results

### 3.1 Morphology diagram of mixtures of the cationic PAs C_16_KK and C_16_KKK

**Fig. 1:**
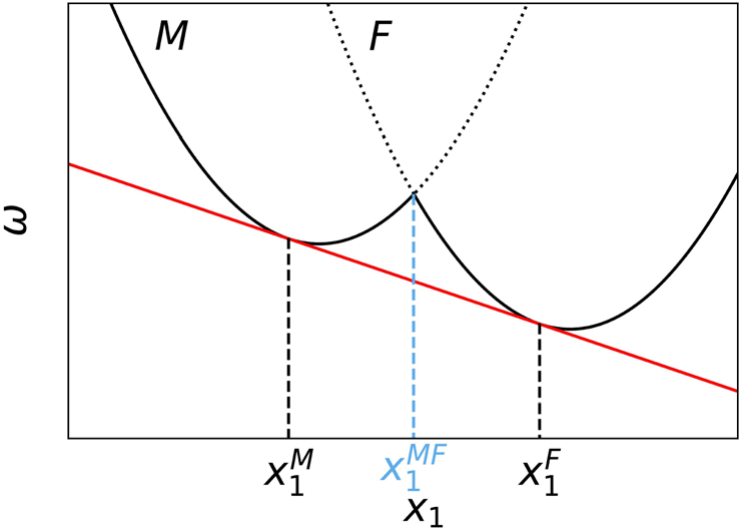
Scheme of the free energy per PA (*ω*) as a function of the molar fraction of PA_1_ in the mixture, *x*_1_, for the spherical micelle (M) and cylindrical nanofiber (F) morphologies. The solid red line is tangent to the M and F curves at points *x*^M^_1_ and *x*^F^_1_, respectively. For all global compositions between *x*^M^_1_ and *x*^F^_1_, there is coexistence between micelles with composition *x*^M^_1_ and fibers with composition *x*^F^_1_.

In some cases, the plots of the minimal free energy *vs.* composition (*ω vs. x*_1_) for the different morphologies have shapes similar to that in the scheme in Fig. 1. This plot indicates that micelles and fibers are the stable morphologies for *x*_1_ < *x*^M^_1_ and *x*_1_ > *x*^F^_1_, respectively. However, for *x*^M^_1_ < *x*_1_ < *x*^F^_1_, the free energy of the system can be lowered through coexistence of micelles with composition *x*^M^_1_ and fibers with composition *x*^F^_1_, see the red line in Fig. 1. This red line is tangent to the M and F curves (solid black curves) at points *x*^M^_1_ and *x*^F^_1_, which establish the criteria used to select these points (see the ESI† for a detailed argument, note also that the points *x*^M^_1_ and *x*^F^_1_ do not necessarily lie at the minima of the curves). This argument to predict morphology coexistence is analogous to that used to explain phase coexistence in systems exhibiting first-order phase transitions, such as phase separation in mixtures of immiscible liquids.^42,43^ However, it is important to mention that this similarity does not imply that morphological transitions in experimental surfactant solutions are first order. Instead, this type of transition arises in our theory from the approximation of considering only aggregates with minimal *ω* and, thus, neglecting the natural size and shape distribution of the micellar aggregates.^25^

**Fig. 2:**
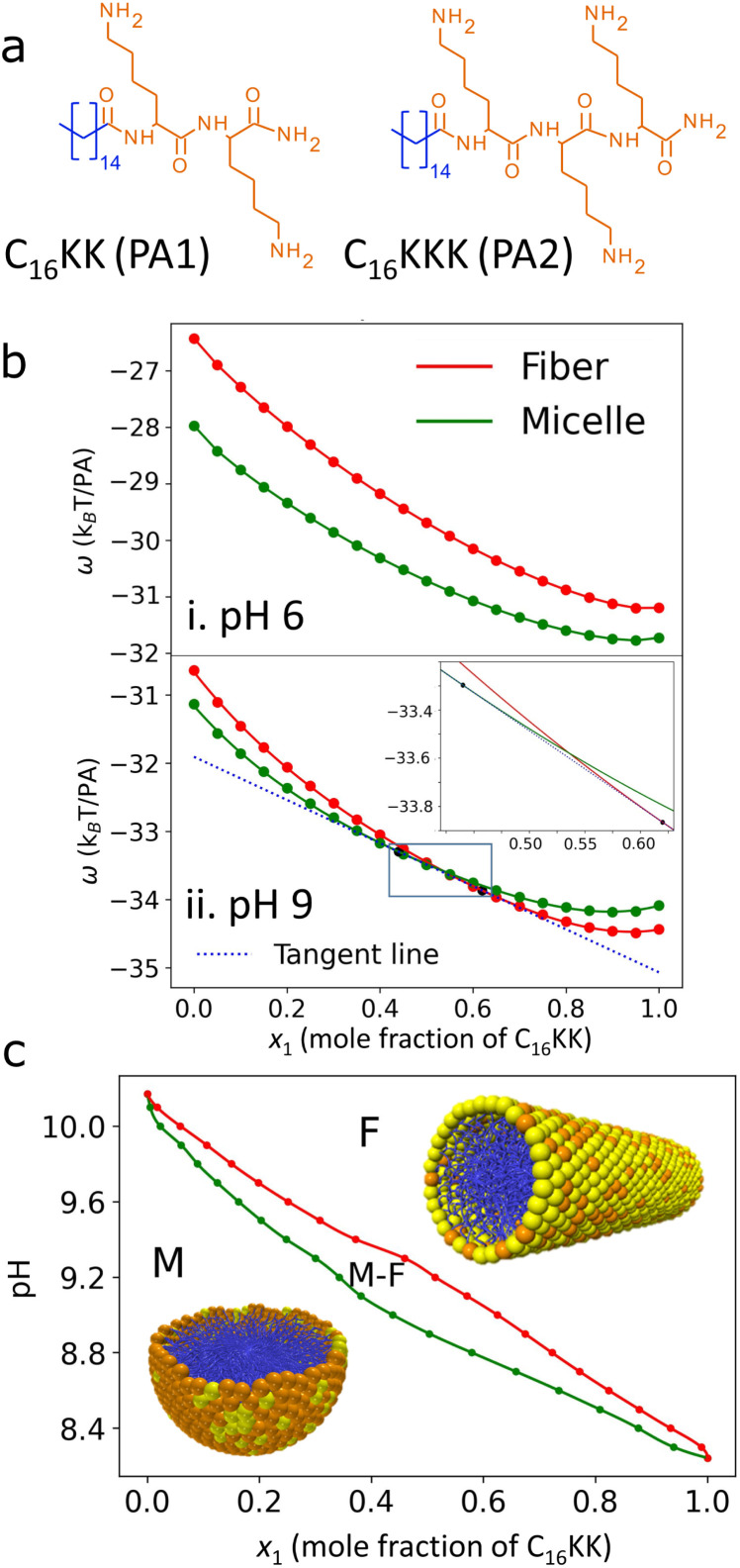
(a) Structures of the PAs used in the C_16_KK/C_16_KKK mixture. (b) Free energy per PA for this mixture at different pH values determined through MOLT calculations assuming ideal cylindrical fibers (red solid lines) or spherical micelles (green solid lines). For pH = 9, the dashed line (tangent to the free-energy curves) indicates micelle–fiber coexistence (the inset shows an enlargement of the region indicated with a box). (c) Predicted morphology diagram. M and F indicate the region where fibers and micelles are stable, respectively, and M–F is the coexistence region.

We start our analysis by studying the theoretical predictions for a mixture of C_16_KK (PA_1_) and C_16_KKK (PA_2_), see structures in Fig. 2a. Individually, both molecules are known to transition from spherical micelles to cylindrical fibers upon increasing the solution pH.^18–20^ This transition results from the deprotonation of lysines at high pH values, which reduces the electrostatic repulsions between the amphiphile head groups, favoring the least curved morphology (fibers). The transition pH is higher for C_16_KKK (pH ∼ 9 (ref. 18)) than for C_16_KK (pH ∼ 7.5 (ref. 18)) because the additional lysine increases the electrostatic and steric repulsions, thereby stabilizing the micelle morphology. On the other hand, both transition pH values are significantly smaller than the p*K*_a_ of the amino group in the lysine side chain (p*K*_a_ = 10.54), but closer to the apparent p*K*_a_s of these amino groups in the nanostructures (p*K*^app^_a_ = 8.4 for C_16_KK and 9.2 for C_16_KKK^18^). These apparent p*K*_a_s are smaller than the p*K*_a_ of the free amino acid because repulsive electrostatic interactions in the assembly decrease the degree of protonation of the amines (*i.e.*, the charge-regulation effect^20^).

In Fig. 2b, we show the two different types of behavior observed for the free-energy *vs.* composition (*x*_1_) curves for this mixture. For pH 6, the free energy curves for fibers and micelles do not intersect and, therefore, the one with the lowest free energy indicates the stable morphology (in this case, micelles) for all compositions. In the second example (pH 9), the free-energy curves intersect and the plot has a similar shape to that in Fig. 1. Thus, there is a M → F transition with its corresponding region of M–F coexistence (this coexistence region is difficult to visualize due to the slope of the curves, see the inset in Fig. 2b(ii)).

We calculated *x*^M^_1_ and *x*^F^_1_ for each pH to construct the pH *vs.* composition morphology diagram shown in Fig. 2c. For all compositions, there is a M → F transition with increasing pH, which is consistent with previous observations for the monocomponent systems. The M ↔ F transition pH values for the pure PAs C_16_KKK (*x*_1_ = 0, pH 10.2) and C_16_KK (*x*_1_ = 1, pH 8.2) also agree with the predictions and experimental observations in our previous work.^20^ In the region enclosed by the two curves, MOLT predicts coexistence between micelles enriched in C_16_KKK and fibers enriched in C_16_KK (the proportion of each morphology in the mixture is given by the lever rule^43^).

**Fig. 3:**
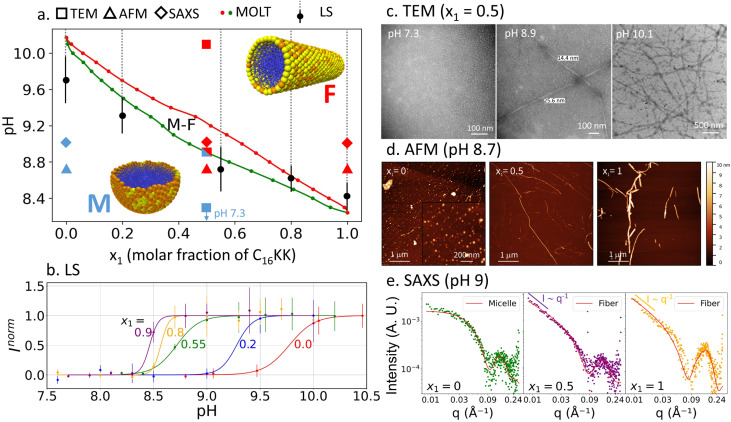
(a) Experimental verification of the morphology diagram of C_16_KK/C_16_KKK mixtures. The figure shows the boundaries for the M → F transition predicted by MOLT (red and green solid circles, same as in Fig. 2c) and measured by LS (solid black circles and dashed gray lines, which indicate regions of high LS). The TEM, AFM and SAXS observations under specific conditions were categorized as micelles (blue symbols), fibers (red) or micelles + fibers (blue and red). (b) LS normalized intensity as a function of pH for mixtures with different ratios of C_16_KK and C_16_KKK. Solid lines show the best fit using the sigmoid function in eqn (1). The LS intensity, *I*, was normalized using the fitting parameters as *I*^norm^ = (*I* − *I*^min^)/(*I*^max^ − *I*^min^). (c) TEM images for *x*_1_ = 0.5 and different pHs. (d) AFM at pH 8.7 and different values of *x*_1_. (e) SAXS curves at pH 9 and different *x*_1_ values.

We tested the theoretical predictions by studying C_16_KK/C_16_KKK mixtures with a combination of experimental techniques: LS, TEM, AFM and SAXS. LS experiments reveled a significant increase in scattering intensity (counts per s) upon increasing the solution pH for a fixed composition (Fig. 3b). This increase corresponds to the threshold of the M → F transition because fibers scatter light more efficiently than micelles. We fitted the LS intensity as a function of pH for mixtures of different composition using a sigmoid function:1
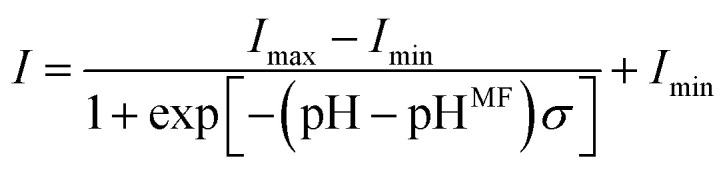
where 1/*σ* is the width of the transition and pH^MF^ is the micelle → fiber transition pH, which we plotted against MOLT predictions in the morphology diagram of Fig. 3a (the dashed lines indicate the region where we measured high LS, indicating the presence of fibers).

Fig. 3c shows TEM images for a mixture of C_16_KK and C_16_KKK with *x*_1_ = 0.5 at different pH values. At pH 7.3, we observe micelles, in line with MOLT predictions (Fig. 3a). At pH 8.9, we observe micelles coexisting with high-aspect ratio objects. This result may indicate the M–F coexistence predicted by MOLT in that region, although we note that the shape and width (15–25 nm) of the elongated objects seem more consistent with planar ribbons or bundles of fibers than with the single cylindrical fibers previously observed for pure C_16_KK^19^ and C_16_KKK.^20^ At pH = 10.1, TEM images show fiber-like aggregates, also in agreement with the predicted morphology diagram. AFM experiments at pH 8.7 (Fig. 3d) reveal that pure C_16_KK and C_16_KKK form well-defined fibers and micelles, respectively, in line with previous observations for these PAs^19,20^ and MOLT predictions. For the *x*_1_ = 0.5 mixture, we observe both long fibers and short objects, which can be either segmented fibers or spherical micelles.

SAXS experiments at pH 9 for *x*_1_ = 0, 0.5, and 1 are shown in Fig. 3e. At low *q* values, the SAXS intensity follows a power-law behavior, *I* ∼ *q*^*α*^. The experimental exponents *α* = −0.15 (for *x*_1_ = 0), *α* = −1.4 (for *x*_1_ = 0.5) and *α* = −1.2 (*x*_1_ = 1.0) are consistent with theoretical expectations: for spherical micelles, the intensity should decay with a slope close to zero, while for elongated cylindrical fibers, a slope near −1 is expected due to their one-dimensional form factor.^19,44^Table 1 summarizes the structural parameters obtained from the fits. The core radii range from 1.6 to 2.1 nm, while the shell thicknesses vary between 0.7 and 1.1 nm. These values are in good agreement with previous reports for the pure PAs^19,41^ and with MOLT predictions (see below).

**Table 1:** Main structural parameters obtained from SAXS curve fitting for C_16_KK/C_16_KKK mixtures at pH 9. The fiber lengths were fixed to 1 μm in all cases

*x*_1_ (fraction of C_16_KK)	Morphology	Core radius (nm)	Shell thickness (nm)
0	Micelle	2.1	1.0
0.5	Fiber	1.6	1.1
1	Fiber	1.7	0.7

The combined experimental and theoretical data in Fig. 3a show that our modeling framework can quantitatively predict the effect of composition on morphology. Furthermore, the results demonstrate that the transition pH can be continuously tuned by varying the composition of the mixture.

### 3.2 Phase behavior of a mixture of oppositely charged peptide amphiphiles

**Fig. 4:**
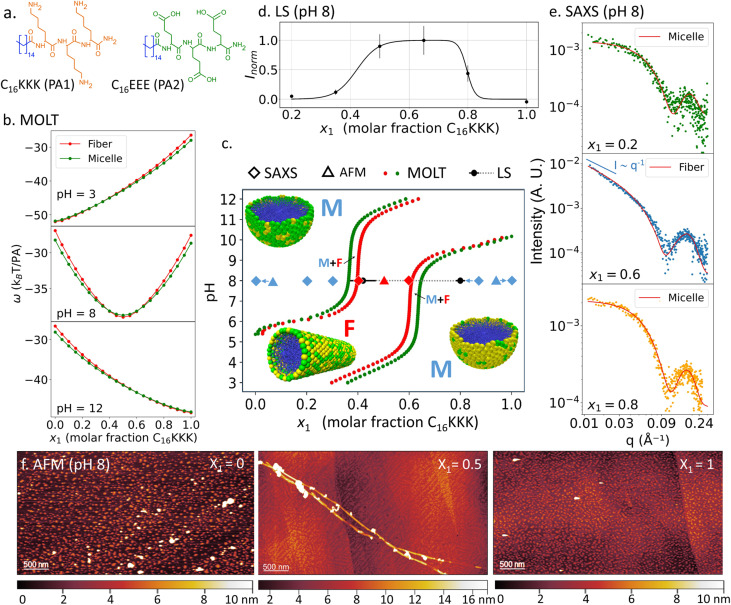
Morphology behavior of C_16_KKK/C_16_EEE mixtures. (a) Structures of the PAs in the mixture. (b) Free energy per PA at different pH values determined through MOLT calculations assuming ideal cylindrical fibers (red solid lines) or spherical micelles (green solid lines). (c) Comparison of the boundaries of the M ↔ F transition predicted by MOLT (solid red and green circles), measured by LS (solid black circles and dashed gray lines, which indicate regions of high LS) and obtained from SAXS experiments, which were categorized as micelles (blue symbols) or fibers (red). (d) Normalized LS intensity *vs. x*_1_ for pH 8. Solid lines show the best fit using a product of two sigmoid functions. (e) SAXS curves at pH 8 and different *x*_1_ values. (f) AFM images at pH 8 and different *x*_1_ values.

Next, we examine a mixture of C_16_KKK and C_16_EEE (E: glutamic acid, see structures in Fig. 4a). MOLT calculations in Fig. 4b reveal that the free-energy curves for fiber and micelle intersect at pH 3 and 12, indicating transitions between them, as well as the corresponding coexistence region. At pH 8, the mixture exhibits a very interesting behavior: the curves intersect at two different points, indicating a M → F → M transition for increasing *x*_1_.

We used MOLT to predict the morphology diagram of the system (Fig. 4c). For *x*_1_ close to 1, the behavior resembles that of pure C_16_KKK, as expected. On the other hand, the morphology behavior of pure C_16_EEE (*x*_1_ = 0) is predicted to be opposite to that of C_16_KKK as it displays a F → M transition with increasing pH. At low pH, the glutamic acid residues are mostly neutral and do not repel electrostatically, resulting in a stable fiber morphology. At high pH, the carboxyl groups of glutamic acid become deprotonated and negatively charged, which favors the formation of micelles because the electrostatic repulsion between headgroups increases the curvature of the assembly. Wester *et al.*^35^ found a transition from elongated objects (classified as ribbons) to micelles at pH ∼ 6.5 for the same molecule. The facts that the transition involves ribbons instead of fibers and occurs at a pH higher than that predicted by MOLT (pH 5.5) indicates that some refinement is needed for the bead–bead interaction parameters in MOLT (for simplicity, we used the same short-range interaction parameters as for C_16_KKK and simply changed the charge and p*K*_a_ of the side chain to model glutamic acid). Note also that the predicted transition pH (pH 5.5) is higher than the p*K*_a_ of a free carboxylic acid (“bulk p*K*_a_”, we used a value of 4.5 in the calculations) because of the charge-regulation effect. As discussed above, for positively charged PAs, the apparent p*K*_a_ is lower than the bulk p*K*_a_ because electrostatic interactions stabilize the neutral species (–NH_2_) over the charged one (–NH_3_^+^). For negatively charged PAs, the stabilization of the neutral state (–COOH) results in an apparent p*K*_a_ that is higher than the bulk p*K*_a_.^45^

At intermediate *x*_1_ values, the electrostatic repulsions between the peptide head groups are reduced because the assembly is approximately charge neutral. This situation stabilizes the fiber morphology. This prediction agrees remarkably well with the observation of fibers by TEM and SEM by Wester *et al.*,^35^ for 1 : 1 C_16_KKK/C_16_EEE mixtures at pH 6.45 and pH 6.9. To further test our theoretical predictions, we conducted a LS experiment at fixed pH 8 and varying ratios of C_16_KKK and C_16_EEE (Fig. 4d). This experiment confirms the presence of a M → F → M transition with increasing *x*_1_ as predicted by MOLT. In this case, we fitted the LS data using a product of sigmoid functions:2
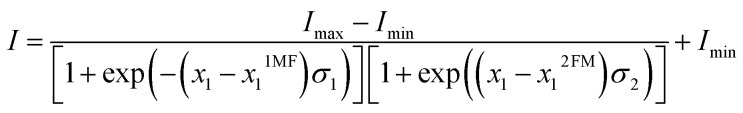
and plotted the transition compositions (*x*_1_^1MF^ = 0.42 ± 0.05 and *x*_1_^2FM^ = 0.8 ± 0.02) in the morphology diagram of Fig. 4c for comparison.

SAXS experiments were performed at pH 8 and different values of *x*_1_ (Fig. 4e and S8 in the ESI†). Table 2 shows the low-*q* exponents and the best fitting parameters using the same core–shell model and SLD values used above for C_16_KK/C_16_KKK mixtures. For *x*_1_ = 0.4 and 0.6, we find *α* close to −1 (−1.1 and −1.4, respectively), indicating a fiber morphology. Furthermore, these curves can be fitted to the core–shell fiber model described above. The samples with *x*_1_ = 0, 0.2, 0.3, 0.8 and 1 show a good fit to the core–shell spherical micelle model, which confirms the existence of a M → F → M transition, in excellent agreement with MOLT and LS. This result is also confirmed by the AFM experiments shown in Fig. 4f.

**Table 2:** Main structural parameters obtained from SAXS curve fitting for a C_16_KKK/C_16_EEE mixture at pH 8

*x*_1_ (fraction of C_16_KKK)	Morphology	Core radius (nm)	Shell thickness (nm)	Total radius (nm)	Fiber length (nm)
0.0	Micelle	0.9	0.9	1.8	—
0.2	Micelle	1.9	1.0	2.9	—
0.3	Micelle	2.0	1.1	3.1	—
0.4	Fiber	1.7	1.1	2.8	17.6
0.6	Fiber	1.5	1.1	2.3	>1000
0.8	Micelle	1.7	0.8	2.5	—
1.0	Micelle	1.7	0.9	2.6	—

**Fig. 5:**
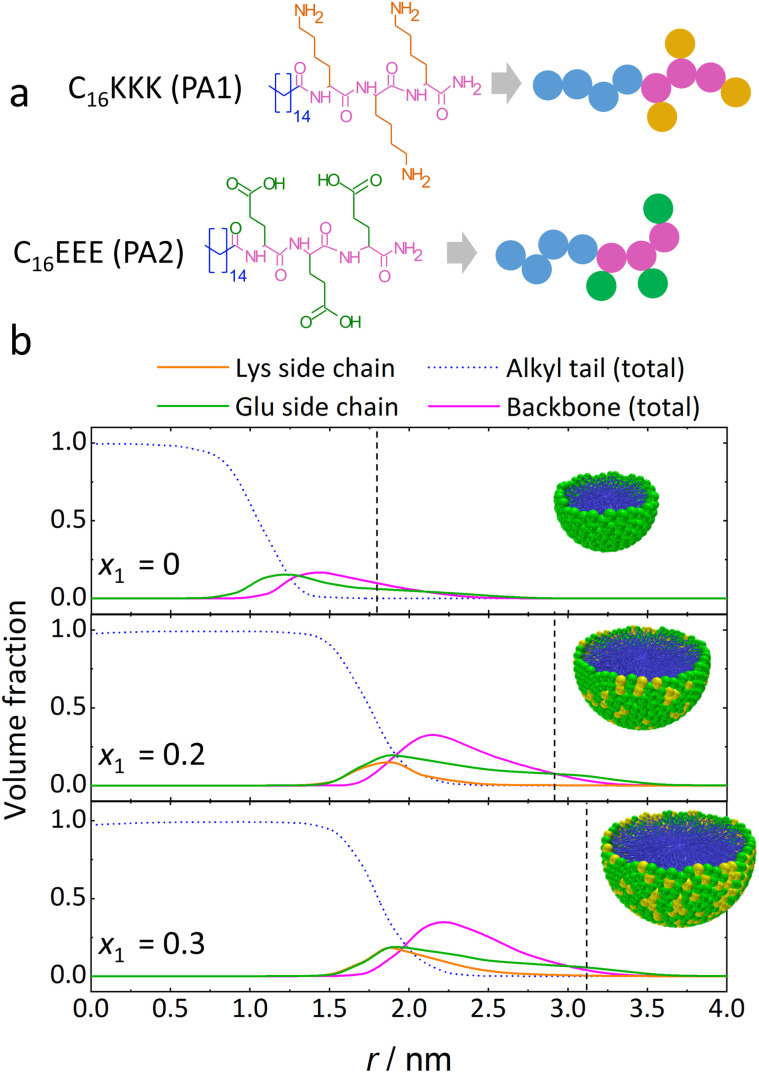
(a) Coarse-grain strategy for C_16_KKK and C_16_EEE used in MOLT calculations. The C_16_ alkyl chain is represented by four alkyl-tail beads (blue). The amino acid backbone (magenta) and side chains (orange for lysines and green for glutamic acid) are represented by a single bead each. (b) Volume fraction of the beads *vs.* distance from the center of a spherical micelle composed of a C_16_KKK/C_16_EEE mixture for different values of *x*_1_ (molar fraction of C_16_KKK).

An interesting observation in Table 2 is that the micelle size increases when *x*_1_ increases from 0 (pure C_16_EEE) to 0.3. In Fig. 5, we compare these SAXS measurements with MOLT predictions. Fig. 5a shows the coarse-grain structure used in MOLT calculations, where each bead represents either four methylenes (tail beads in blue), the backbone atoms of an amino-acid (magenta) or its side chain (orange for lysine, green for glutamic acid). Fig. 5b shows the predicted volume fraction of each of these beads as a function of the distance from the micelle center, *r*, for different values of *x*_1_. As expected,^20,46^ tail beads form the core of the nanostructure, while the amino acids are located in the outer region. Comparing SAXS radii with MOLT predictions is not straightforward because the micelle/solvent interface is not a sharp step function, as assumed in SAXS modeling. However, it is noteworthy that the decay of the volume fraction profiles predicted by MOLT occurs near the experimental SAXS radii (shown by vertical dashed lines in Fig. 5b). Also, MOLT shows a marked increase in micellar size from *x*_1_ = 0 to *x*_1_ = 0.2, in line with SAXS results, which we ascribe to a decrease in electrostatic repulsions as the mixtures approach charge neutrality. Finally, the radii of the hydrophobic core predicted by MOLT and measured by SAXS are always smaller than the length of a fully stretched C_16_ chain (∼1.92 nm (ref. 47)), while the length of a tripeptide (∼0.8–1.1 nm (ref. 48)) is similar to the shell thicknesses measured by SAXS and of the corona region predicted by MOLT. The height of the micelles observed by AFM in Fig. 4f (∼4.5 nm for C_16_EEE and ∼3 nm for C_16_KKK) is comparable to twice the radii measured by SAXS and predicted by MOLT (in the case of C_16_KKK, the height is slightly smaller than that expected from the radius, which may be attributed to micelle deformation on the substrate).

**Fig. 6:**
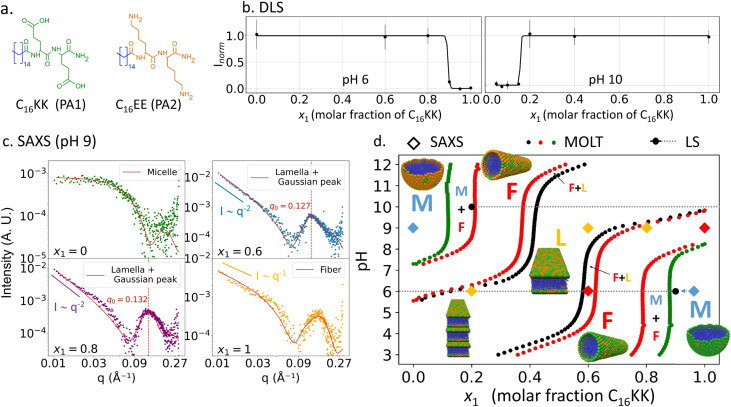
Morphology behaviour of C_16_KK/C_16_EE mixtures. (a) Structures of the PAs used in the mixtures. (b) Normalized LS intensity *vs. x*_1_ at pH values, 6 and 8. Solid lines show the best fit using a sigmoid function. (c) SAXS curves at pH 9 and different *x*_1_ values. (d) Comparison of the boundaries of the M ↔ F transition predicted by MOLT (solid red, green and black circles) and measured by LS (solid gray circles and dashed gray lines, which indicate regions of high LS) and SAXS experiments for specific conditions that were categorized as micelles (blue symbols), fibers (red) or fibers + lamellae (red and yellow). The symbols M, F and L indicate the regions of stability for micelles, fibers and lamellae, respectively.

We also studied another mixture of oppositely charged PAs, the C_16_KK/C_16_EE system (structures shown in Fig. 6a). An interesting prediction from MOLT is that this mixture forms planar lamellae under some conditions (see the morphology diagram in Fig. 6d). Note that in all systems discussed above, the free energy of the lamellar morphology was higher than that of fibers and/or micelles (pure C_16_KK is predicted to form lamellae at pH > 10.2,^20^ just above the range of pH studied in Fig. 2). The predicted morphology diagram of C_16_KK/C_16_EE is similar to that in Fig. 4c for C_16_KKK/C_16_EEE, with the difference that it shows a lamellar morphology for nearly stoichiometric mixtures and, therefore, it displays L → F and F → L transitions with increasing *x*_1_, in addition to the M → F and F → M transitions already predicted for C_16_KKK/C_16_EEE.

LS experiments for C_16_KK/C_16_EE mixtures (Fig. 6b) show that increasing the content of C_16_KK leads to an elongated object → M transition at pH 6, but results in an M → elongated object transition at pH 8. Note that LS does not distinguish between fibers and lamellae (*i.e.*, planar ribbons with a bilayer structure^19^) because both greatly increase the scattering with respect to the micelles. AFM experiments (Fig. S8 in the ESI†) also indicate the formation of elongated objects forming a network. However, these experiments are inconclusive about whether these aggregates are narrow planar ribbons, bundles of fibers or a combination of both.

SAXS experiments on C_16_KK/C_16_EE mixtures at pH 9 (Fig. 6d and Table 3) show micellar structures for *x*_1_ = 0 and fibers for *x*_1_ = 1. For *x*_1_ = 0.6 and 0.8, the low-*q* exponents are close to −2.0 (−2.0 and −1.7, respectively), which are values characteristic of lamellar assemblies.^19^ These SAXS curves were best fitted using a combination of a lamellar ribbon model and a broad Gaussian peak centered at high *q* values (see the ESI† for details). This Gaussian peak arises from correlations between stacked lamellar units. The center of the Gaussian peak is located at *q*_0_ = 0.127 Å^−1^ for *x*_1_ = 0.6 and *q*_0_ = 0.132 Å^−1^ for *x*_1_ = 0.8, corresponding to characteristic distances of *d* = 2π/*q*_0_ = 4.94 nm and 4.76 nm, respectively. The characteristic distances refer to the inter-lamellar spacing—that is, the average center-to-center distance between adjacent lamellar domains. The presence of lamellae at *x*_1_ = 0.6 and 0.9 and pH 9 is in agreement with MOLT predictions. While MOLT captures the overall morphological transition, SAXS provides additional structural insight by revealing the presence of ordered lamellae forming short-range domains (*i.e.*, a small number of stacked lamellar units), as evidenced by the broadness of the fitted peaks in the lamellar phase. Additional SAXS measurements at pH 6 were also performed and are shown in Fig. S10 of the ESI.† The sample at pH 6 and *x*_1_ = 0.2 also exhibits a correlation peak; however, this peak is sharper than those observed at pH 9, indicating the formation of large domains of stacked lamellae. The presence of the correlation peak due to stacking is strong evidence that the elongated objects observed by LS, AFM and SAXS in these samples have an internal lamellar structure.

**Table 3:** Main structural parameters and low-*q* exponent obtained from SAXS curve fitting for C_16_KK/C_16_EE mixtures

*x*_1_	pH	Morphology	Micelle or fiber		Lamella			Lamella or fiber
			Core radius (nm)	Shell thickness (nm)	Core thickness (nm)	Shell thickness (nm)	Lamella width (nm)	Length (nm)
0.0	9	Micelle	1.3	1.2	—	—	—	—
0.6	9	Lamella	—	—	1.7	1.0	100	>1000
0.8	9	Lamella	—	—	1.4	0.8	100	>1000
1.0	9	Fiber	1.7	0.7	—	—	—	>1000
0.6	6	Fiber	1.5	0.9	—	—	—	291
0.9	6	Micelle	1.5	0.8	—	—	—	—

## 4 Conclusions

In this work, we demonstrated that the composition of peptide amphiphile co-assemblies is a flexible approach to control the morphology and size of their nanostructures. We explored three specific systems. In mixtures of C_16_KK/C_16_KKK, we identified a micelle ↔ fiber transition induced by changes in the solution pH and the composition of the system. In this case, the transition pH can be finely controlled between that of pure C_16_KK and pure C_16_KKK by adjusting system composition. For mixtures of oppositely charged PAs, we observed even more interesting morphological behaviors such as a micelle ↔ fiber ↔ micelle transition upon varying the concentration at a fixed pH (for C_16_KKK/C_16_EEE), as well as lamella ↔ fiber and fiber ↔ lamella transitions (for C_16_KK/C_16_EE). In these cases, the neutralization of the acidic and basic PAs reduces electrostatic repulsions in the system, favoring the formation of lamellae and fibers (the least curved structures) over micelles. This decrease in electrostatic repulsions also explains why the size of micelles increases (*i.e.*, the curvature decreases) when the solution composition approaches charge neutrality.

We showed that MOLT, a molecular theory originally introduced to model the behavior of pure C_16_KK and C_16_KKK,^20^ provides accurate predictions for PA mixtures as well. These predictions are in agreement with most LS, SAXS, AFM and TEM experimental observations, although there are minor discrepancies in specific cases. We ascribe these discrepancies to the approximations inherent in our theoretical framework (such as its coarse-grained nature and the mean-field approximation). In this regard, our theory allows construction of morphology diagrams directly from the free energies of the aggregates, which are predicted at a computational cost much smaller than that of atomistic MD simulations (for example, a recent study using atomistic MD was limited to the self-assembly of clusters of a few PAs^23^). A novel theoretical prediction resulting from this work is the coexistence of different morphologies near structural transitions. In the coexistence region, each morphology is enriched in a different PA. TEM and SAXS provide experimental evidence for micelle–fiber (Fig. 2c) and fiber–lamella (Fig. 6c) coexistence, respectively.

In summary, we demonstrated that controlling the composition of PA co-assemblies is a powerful strategy for tuning the morphology and size of their aggregates. An interesting question that needs to be addressed in the future is whether the functional properties of these co-assemblies (such as their antimicrobial activity^13,18,49,50^) can also be finely tuned by varying their composition.

## Author contributions

M. F., M. C. S. and M. T. designed the work and planned the experiments. M. F. and M. T. performed the theoretical calculations. H. X. and M. C. S. synthesized the PAs. Characterization experiments were performed by M. F. (AFM, LS, and SAXS), H. X. (TEM), M. C. (SAXS), O. G. (LS), S. H. (AFM), A. P. (SAXS) and G. Y. (LS). M. F. and M. T. wrote the first draft. All authors discussed and revised the manuscript.

## Conflicts of interest

There are no conflicts to declare.

## Supplementary Material

SC-016-D5SC02935J-s001

## Data Availability

Data are available from the authors on reasonable request.
