# Supplementary material for: Fine tuning the morphology of peptide amphiphile nanostructures via co-assembly
Source: Chem Sci. 2025 Jul 3;16(32):14605–15. doi: 10.1039/d5sc02935j (PMC12257164; doi:10.1039/d5sc02935j)
Supplement: SC-016-D5SC02935J-s001 [file SC-016-D5SC02935J-s001.pdf]

Supplementary Information (SI) for Chemical Science.
This journal is © The Royal Society of Chemistry 2025

SI for:

Fine tuning the morphology of peptide-amphiphile nanostructures via co-assembly

Maria Mercedes Fiora, Huihua Xing, Marilina Cathcarth, Octavio Garate, Santiago Herrera, Agustin S. Picco, Gabriel Ybarra, Martin Conda-Sheridan*, Mario Tagliazucchi*

E-mail: mario@qi.fcen.uba.ar;martin.condasheridan@unmc.edu

MALDI and NMR characterization of PAs

We present below the ^{1}H NMR and MALDI characterization of all PAs used in this work.

For NMR, all samples were run in deuterated DMSO (d6-DMSO) with a reference peak at 2.49 ppm. The water (H_2O, HDO) peak appears at 3.34 ppm. The most relevant peaks of each molecule are assigned. Amine and carboxylic acid peaks are not seen or present lower integrations due to exchange with deuterium. Note that the NMR spectra of PAs is complex to analyze for several reasons, such as the possibility of self-assembly or aggregation in the deuterated solvent, the multiple exchangeable protons (which can affect the integration values and splitting patterns) and their broad signal, intra- and inter-molecular hydrogen bonds (broaden the peaks) and the overlap of peaks.

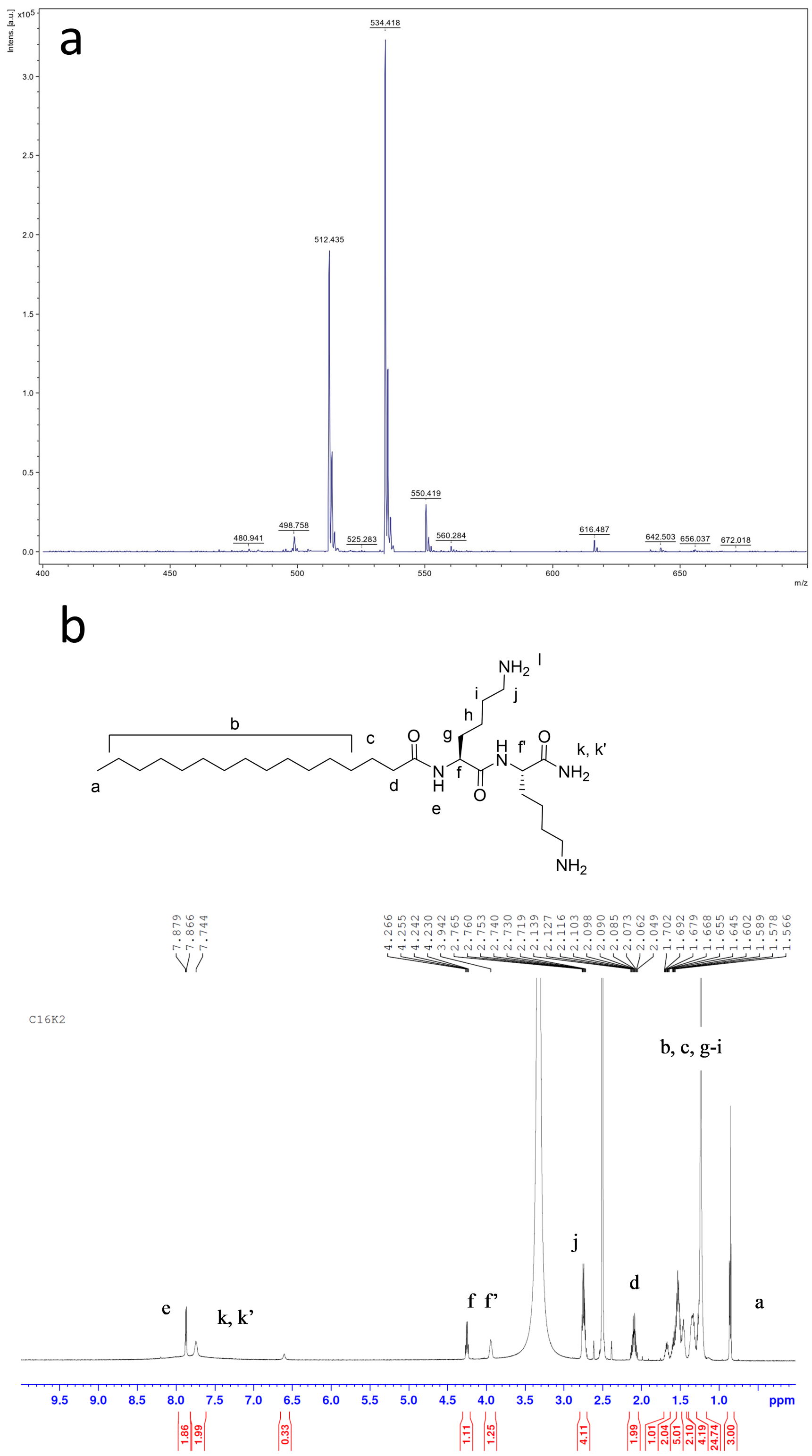

Figure S1: a. MALDI spectrum of $C_{16}KK$. Calculated Mw (g/mol): $C_{16}KK$: 511.78 ($C_{28}H_{57}N_5O_3$), $C_{16}KK+Na$: 534.78, $C_{16}KK+K$ (potassium): 550.88. b. ^1H NMR of $C_{16}KK$.

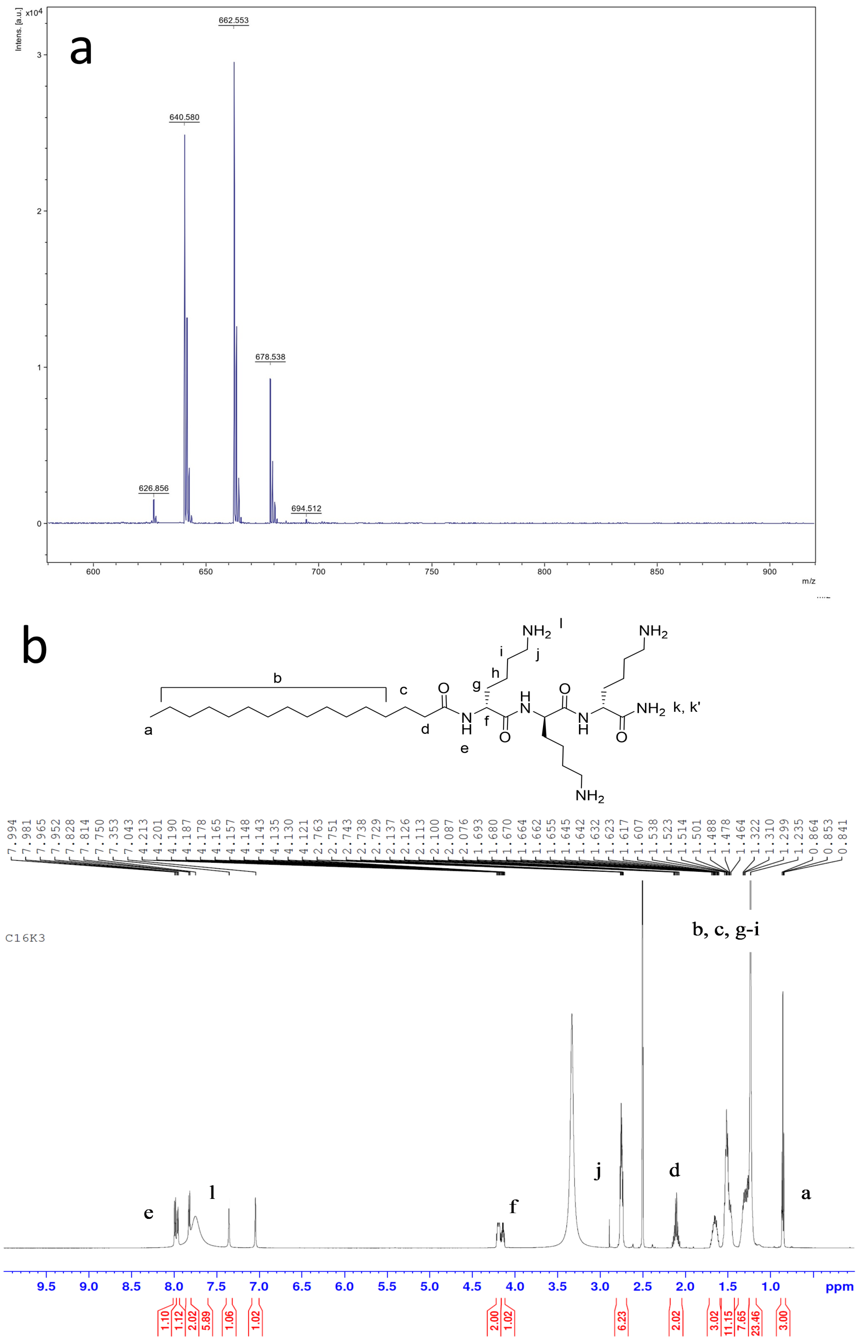

Figure S2: MALDI spectrum of C_{16}KKK. Calculated Mw: (g/mol) C_{16}KKK: 639.96 ($C_{34}H_{69}N_7O_4$), C_{16}KKK+Na: 662.96, C_{16}KKK+K (potassium): 679.06. b. ^{1}H NMR of C_{16}KKK.

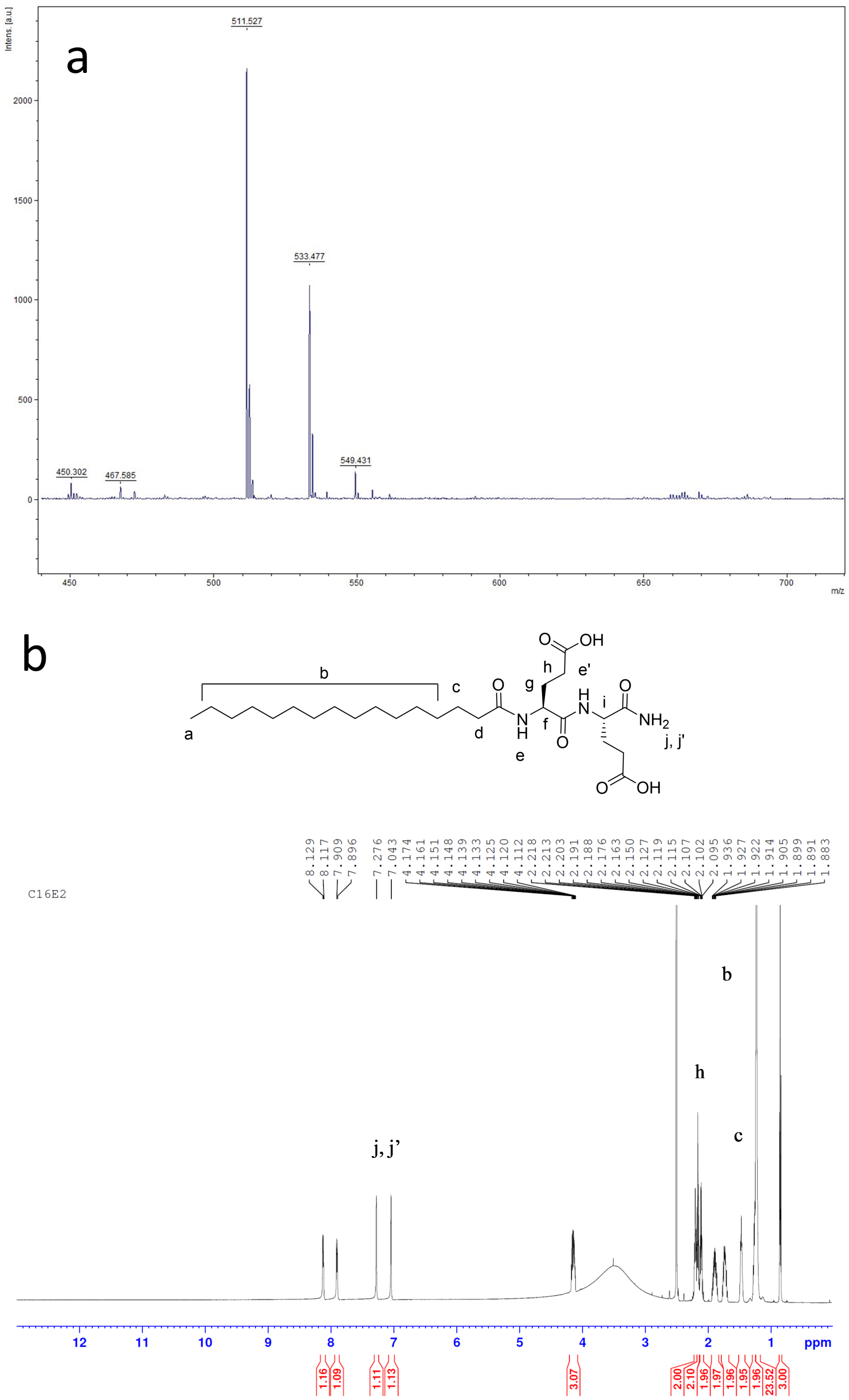

Figure S3: MALDI spectrum of C_{16}EE. Calculated Mw (g/mol): C_{16}EE: 513.68 ($C_{26}H_{47}N_3O_7$), C_{16}EE+Na: 536.68, C_{16}EE+K (potassium): 552.68. b. ^{1}H NMR of C_{16}EE.

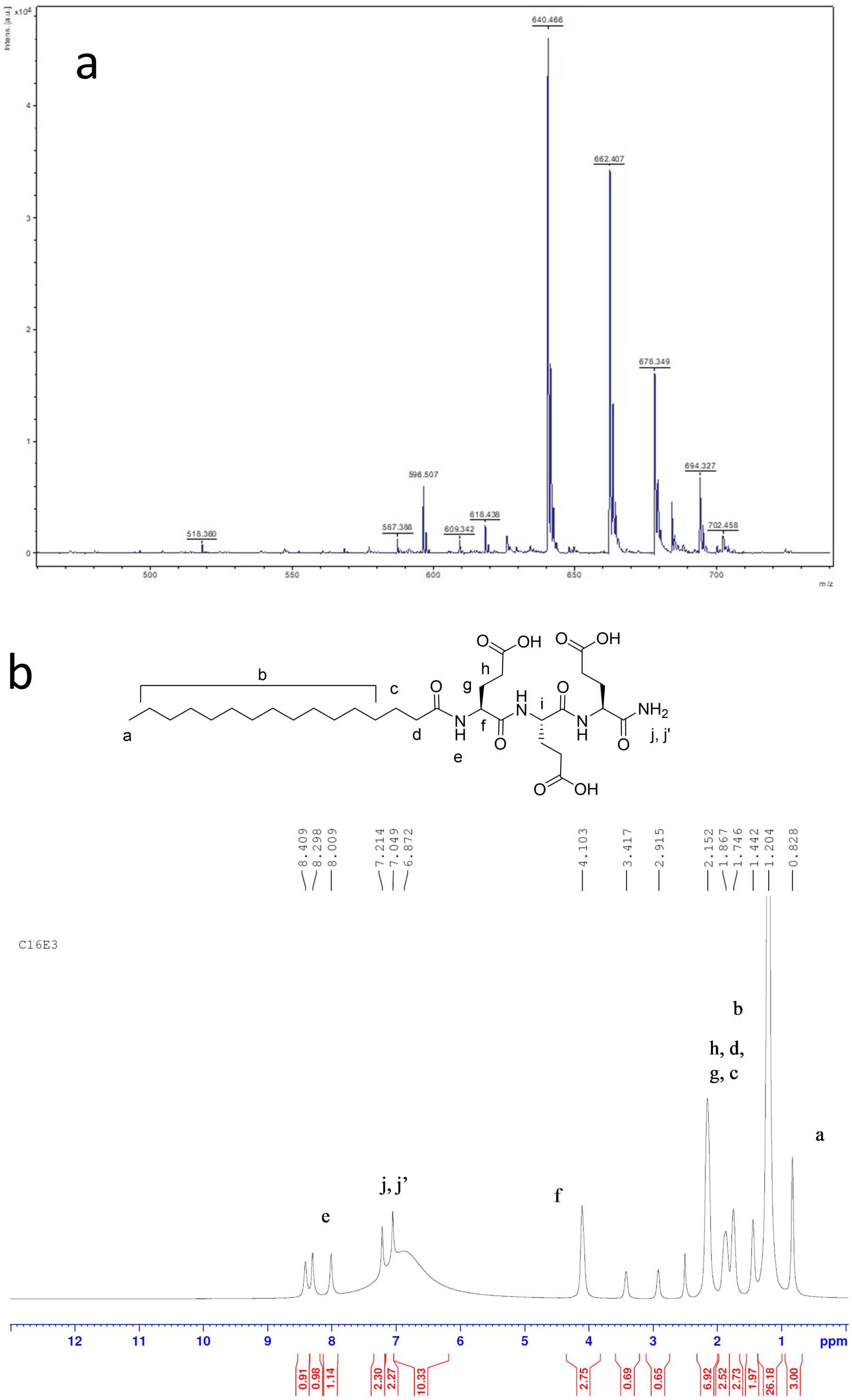

Figure S4: MALDI spectrum of C_{16}EEE. Calculated Mw (g/mol): C_{16}EEE: 642.79 ($C_{31}H_{54}N_4O_{10}$), C_{16}EEE+Na: 665.79, C_{16}EEE+K (potassium): 681.79. b. ^{1}H NMR of C_{16}EEE.

Theoretical Methods

Free-energy functional

Our theoretical framework is grounded in a molecular theory (MOLT) developed by Szleifer and colleagues to model soft materials.[1] In prior studies, we adapted this theory to investigate the self-assembly of (single-component) peptide-amphiphile (PA) nanostructures,[2] as well as mixtures of simple surfactants and non-polar additives.[3,4] In this section, we present the derivation of the theoretical approach to describe co-assemblies involving multiple PAs. The methodology involves formulating an approximate semi-grand canonical free-energy functional and determining its extremum with respect to the functions that characterize the system's structure. We consider an aggregate consisting of M different amphiphilic components in a water solution with a fixed salt concentration and pH. The number of molecules of the PA of type i (denoted as PA_i) in the aggregate is n_i (note that while the calculations presented in the main text correspond to M=2, we derive here the most general case for an arbitrary number of components). In our theoretical model, the aggregate is assumed to be spatially fixed, meaning its translational entropy is not accounted for in the calculations. The total Helmholtz free energy of the aggregate is expressed as:

$$\beta F^* = -\frac{S_{translational}}{k_B} - \frac{S_{conformational}}{k_B} + \beta F_{interactions} - \frac{S_{mix,chem}}{k_B} + \beta F_{chemical} + \beta F_{electrostatic} \tag{1}$$

where $\beta = (k_B T)^{-1}$, k_B is Boltzmann's constant and T is the absolute temperature. The superscript * in F^* indicates that the free energy corresponds to an aggregate fixed in space.[2,5]

The first term on the right-hand side of equation (S1) represents the free-energy contribution from the translational entropy of all species within the aggregate, including salt ions, protons, hydroxyl ions, PA molecules, and solvent. This term is calculated as follows:

$$-\frac{S_{translational}}{k_B} = \sum_{\substack{\text{i= PA}_1,\dots,\text{PA}_M, \\ \text{anion,cation,H}^+,\text{OH}^-,\text{sol}}} \int \rho_i(\mathbf{r}) \left[\ln\left(\rho_i(\mathbf{r}) v_{sol}\right) - 1\right] d\mathbf{r} \tag{2}$$

where $\rho_i(\mathbf{r})$ is the number density of species i, for i = PA molecules, solvent, salt cation, salt anion, H^+ or OH^- at position $\mathbf{r}$. v_{sol} is the molecular volume of the solvent. The second term in equation 1 is the free energy associated with the PA conformational entropy:

$$-\frac{S_{conformational}}{k_B} = \sum_{\text{k= PA}_1,\dots,\text{PA}_M,} \int \rho_k(\mathbf{r}) \sum_{\alpha} P_k(\alpha, \mathbf{r}) \ln\left(P_k(\alpha, \mathbf{r})\right) d\mathbf{r} \tag{3}$$

In this equation, $P_k(\alpha, \mathbf{r})$ is the probability of having a molecule of PA_k in the conformation α when its center of mass is at $\mathbf{r}$. The sum over α runs over the conformations of the molecule of type k.

The next term in the free-energy functional represents the effective bead-bead non-electrostatic interactions:

$$\beta F_{interactions} = -\frac{1}{2} \sum_{\substack{i=\text{all}\\ \text{bead types}}} \sum_{\substack{j=\text{all}\\ \text{bead types}}} \int\int \langle n_i(\mathbf{r})\rangle \langle n_j(\mathbf{r}')\rangle \beta\varepsilon_{ij} g_{ij}(\mathbf{r},\mathbf{r}')\mathrm{d}\mathbf{r}\mathrm{d}\mathbf{r}' \quad (4)$$

In 4, $\langle n_i(\mathbf{r})\rangle$ represents the average number density of beads of type i at position $\mathbf{r}$. In this term, ϵ_{ij} is the interaction parameter that determines the strength of the attractive interactions between a bead of type i and a bead of type j, and $g_{ij}(\mathbf{r},\mathbf{r}')$ is a function that accounts for the geometric dependence of the interactions. It is important to note that this term does not include the steric repulsions between beads, which are described below. The average number of segments $\langle n_i(\mathbf{r})\rangle$ is given by:

$$\langle n_i(\mathbf{r})\rangle = \sum_{\text{k= PA}_1,\ldots,\text{PA}_M} \sum_{\alpha} \int n_i^k(\mathbf{r};\alpha,\mathbf{r}')P_k(\alpha,\mathbf{r}')\rho_k(\mathbf{r}')\,\mathrm{d}\mathbf{r}' \quad (5)$$

Here $n_i^k(\mathbf{r};\alpha,\mathbf{r}')d\mathbf{r}$ is the number of beads of type i that the PA molecule k with its center of mass at position **r**' and in conformation α has in the volume between **r** and **r** + d**r**. The functions $n_i^k(\mathbf{r};\alpha,\mathbf{r}')d\mathbf{r}$ encode the spatial distribution of all segments of all molecules in all conformations considered in the calculations. They are an input to the theory, which is constructed during chain generation, see below.

The fourth term in equation 1 is the free energy associated with the mixing entropy of the species related by the acid-base equilibria. The beads can be either neutral (e.g., alkyl tail and aminoacid backbone beads), acidic (side chain of glutamic acid) or basic (side chain of lysine). Acid segments (HA) are proton donors and their acid-base equilibrium is given by:

$$\mathrm{HA} \longleftrightarrow \mathrm{A}^- + \mathrm{H}^+ \qquad k_\mathrm{a} = \frac{[\mathrm{A}^-]\,[\mathrm{H}^+]}{[HA]} \quad (6)$$

In this equation, k_a is the acid-base equilibrium constant for the deprotonation of HA, and [A$^-$], [H$^+$] and [HA] are the molar concentrations of the species. In analogy, the acid-base equilibrium of basic segments (B) is given by:

$$\mathrm{B} + \mathrm{H_2O} \longleftrightarrow \mathrm{BH}^+ + \mathrm{OH}^- \qquad k_b = \frac{[\mathrm{BH}^+]\,[\mathrm{OH}^-]}{[\mathrm{B}]} \quad (7)$$

The presence of different chemical states for the same group introduces a mixing entropy term, given by:

$$-\frac{S_{mix,chem}}{k_B} = \sum_{\substack{i=\text{ type of}\\ \text{acid-based bead}}} \int \langle n_i(\mathbf{r})\rangle\,[f_i(\mathbf{r})\ln f_i(\mathbf{r}) + (1-f_i(\mathbf{r}))\ln(1-f_i(\mathbf{r}))]d\mathbf{r} \quad (8)$$

where $f_i(\mathbf{r})$ is the average fraction of acid-base beads of type i that are charged at position $\mathbf{r}$. Note that the sum over i includes different types of acid-base beads (both acidic or basic), which are defined by their bulk acidity constant k_a (for acid segments) or basicity constant, k_b (for basic segments).

In the following term of the free energy functional, we include the contributions to the free energy from the standard chemical potentials of the different chemical species in the system. In principle, this term includes contributions from all chemical species, but only those from species participating in acid-base chemical equilibria (protons, hydroxyl ions, charged and uncharged acid-base groups) have thermodynamic consequences and, therefore, are explicitly included. Hence, this term is given by:

$$\beta F_{chem} = \sum_{i=\text{ H}^+,\text{OH}^-} \int \rho_i(\mathbf{r})\beta\mu_i^0 d\mathbf{r} \tag{9}$$

$$+ \sum_{\substack{i=\text{ type of}\\ \text{acid-base bead}}} \int \langle n_i(\mathbf{r})\rangle \left[f_i(\mathbf{r})\beta\mu^0_{i\text{ (charged)}} + (1-f_i(\mathbf{r}))\,\beta\mu^0_{i\text{ (uncharged)}}\right] d\mathbf{r} \tag{10}$$

$$\tag{11}$$

where $\mu_\mathrm{i}{}^0$ is the standard chemical potential of the species i . In particular, $\mu_\mathrm{i}{}^0{}_{\text{(charged)}}$ is the standard chemical potential of a charged bead of type i (BH^+ or A^-) and $\mu_\mathrm{i}{}^0{}_{\text{(uncharged)}}$ is the standard chemical potential of an uncharged bead of type i (B or HA).

The last term in equation 1 results from the electrostatic contribution to the free energy:

$$\beta F_{electrostatic} = \beta \int \left[\langle\rho_Q(\mathbf{r})\rangle\,\psi(\mathbf{r}) - \frac{\varepsilon(\mathbf{r})}{2}[\nabla\psi(\mathbf{r})]^2\right] \mathrm{d}\mathbf{r} \tag{12}$$

In this equation, $\psi(\mathbf{r})$ is the electrostatic potential, $\langle\rho_Q(\mathbf{r})\rangle$ is the average charge density and $\varepsilon(\mathbf{r})$ is the dielectric permittivity at $\mathbf{r}$. ρ_Q is given by:

$$\langle\rho_Q(\mathbf{r})\rangle = \sum_{i=\text{ ions}} \rho_i(\mathbf{r})q_i + \sum_{j=\text{ acid-base beads}} \langle n_j(\mathbf{r})\rangle\, f_j(\mathbf{r})q_j \tag{13}$$

where q_i is the charge of the ion i (in units of elemental charge) and q_j is the charge of the acid-base bead j in its charged state.

The position-dependent dielectric permittivity is modeled as the weighted average of the permittivity of each species:

$$\varepsilon(\mathbf{r}) = \sum_{\substack{\text{i= all bead}\\ \text{types}}} \varepsilon_i\,\langle n_i(\mathbf{r})\rangle\, v_i + \sum_{i=\text{ions,solvent}} \varepsilon_i\rho_i(\mathbf{r})v_i \tag{14}$$

where v_i is the volume of the solvent molecules, the ions (salt cation and anion, H^+ and OH^-) or the bead of type i (see Coarse-graining Strategy in the Molecular Model below).

Assuming that the permittivity of the hydrated ions is equal to that of the solvent, that all beads in the PAs share the same permittivity, and using the fact that the sum of all volume fractions at a given position equals unity (see the packing constraint below), we rewrite $\varepsilon(\mathbf{r})$ as a function of the volume fraction of the PA beads:

$$\varepsilon(\mathbf{r}) = \varepsilon_{PA}\left(\sum_{\substack{k=\text{ all bead}\\ \text{types}}} \langle n_k(\mathbf{r})\rangle\, v_k\right) + \varepsilon_{sol}\left(1 - \sum_{\substack{k\,=\text{ all bead}\\ \text{types}}} \langle n_k(\mathbf{r})\rangle\, v_k\right) \tag{15}$$

In this equation, ε_{PA} is the dielectric constant of the PA beads (*i.e.*, the organic species in the system) and ε_{sol} is the dielectric constant of the solvent and hydrated ions.

The functional F^* is a Helmholtz free energy, which is canonical in all species. However, we are interested in a system where fluctuations in the total number of salt ions are controlled by their chemical potentials, which are determined by the composition of the salt solution and pH in equilibrium with the aggregate. Therefore, we consider a semi-grand canonical potential in which we fix the numbers of the different PA molecules within the aggregate and the chemical potential of the mobile ions (instead of their numbers). This thermodynamic potential is:

$$\beta\Omega^* = \beta F^* - \beta\mu_{cation}\int \rho_{cation}(\mathbf{r})d\mathbf{r} - \beta\mu_{anion}\int \rho_{anion}(\mathbf{r})d\mathbf{r} \tag{16}$$

$$-\beta\mu_{\mathrm{H}^+}\int\left[\rho_{\mathrm{H}^+}(\mathbf{r}) + \sum_{\substack{i=\text{type of acid}\\ \text{bead}}} (1 - f_i(\mathbf{r}))\langle n_i(\mathbf{r})\rangle\right]d\mathbf{r} \tag{17}$$

$$-\beta\mu_{\mathrm{OH}^-}\int\left[\rho_{\mathrm{OH}^-}(\mathbf{r}) + \sum_{\substack{i=\text{ type of basic}\\ \text{bead}}} (1 - f_i(\mathbf{r}))\langle n_i(\mathbf{r})\rangle\right]d\mathbf{r} \tag{18}$$

where μ_i is the chemical potential of the species i. Note that in the $-\mu N$ terms for the protons and the hydroxyl ions, we include both the ions free in solution (first terms inside the brackets) and those bound to acid and basic beads (second terms inside the brackets), respectively.

Extremization of the free-energy functional

In order to solve the molecular theory, we find the functional extremum of the free-energy functional with respect to $\rho_i(\mathbf{r})$, $\psi(\mathbf{r})$, $P_k(\alpha,\mathbf{r})$ (for k = PA_1,...,PA_M), and $f_i(\mathbf{r})$. This extremum is subjected to four restrictions. First, the probability-distribution functions $P_k(\alpha,\mathbf{r})$ are normalized for each PA and at each position $\mathbf{r}$:

$$\sum_{\alpha} P_k(\alpha,\mathbf{r}) = 1; \qquad \forall\mathbf{r} \tag{19}$$

The second constraint enforces global electroneutrality:

$$\int \langle\rho_Q(\mathbf{r})\rangle\, \mathrm{d}\mathbf{r} = 0 \tag{20}$$

The third restriction is a packing constraint that models the repulsive intermolecular

interactions:

$$\sum_{\substack{i=\text{all bead types,}\\ \text{ions,solvent}}} \langle n_i(\mathbf{r})\rangle v_i = 1; \qquad \forall \mathbf{r} \tag{21}$$

The last constraint enforces the integral of the density of PA molecules of each type to be equal to their total number:

$$n_k = \int \rho_{PA_k}(\mathbf{r}) d\mathbf{r} \tag{22}$$

These four constraints are enforced by the use of Lagrange multipliers:

$$L = \beta\Omega^* + \int \beta\pi(\mathbf{r}) \left(\sum_{\substack{i=\text{all bead types,}\\ \text{ions,solvent}}} \langle \phi_i(\mathbf{r})\rangle - 1 \right) \mathrm{d}\mathbf{r} + \beta\gamma \int \langle \rho_Q(\mathbf{r})\rangle \, \mathrm{d}\mathbf{r} \tag{23}$$

$$+ \sum_{k=\mathrm{PA}_1,\ldots,\mathrm{PA}_M} \left\{ \int \lambda_k(\mathbf{r})\rho_k(\mathbf{r}) \left[\sum_\alpha P_k(\alpha, \mathbf{r}) - 1 \right] \mathrm{d}\mathbf{r} + \beta\mu_k^* \left(n_k - \int \rho_k(\mathbf{r}) d\mathbf{r} \right) \right\} \tag{24}$$

In this equation, $\beta\pi(\mathbf{r})$ is the Lagrange multiplier that enforces the packing constraint, Eq. 21, whose physical meaning is that of a local osmotic pressure.[1] μ_k^* (enforcing the integral of the PA densities, Eq. 22) is the standard chemical potential of the PA molecules of type k within an aggregate fixed in space in an infinitely diluted solution. The normalization of the probability distribution functions, Eq. 19, is enforced by the Lagrange multiplier $\lambda_k(\mathbf{r})$, which is related to the single-chain internal partition function of the PA of type k (see below). Finally, γ is the Lagrange multiplier guaranteeing global electroneutrality (Eq. 20) and it is an additive constant to the electrostatic potential, ψ. Since γ can be included in the arbitrary choice of the zero of the electrostatic potential, it will be omitted hereafter.

The functional extremum of $\mathcal{L}$ with respect to $\rho_i(\mathbf{r})$, $\psi(\mathbf{r})$, $P_k(\alpha, \mathbf{r})$, and $f_i(\mathbf{r})$ results in a set of coupled integro-differential equations. We describe below their final expressions (after re-arragement).

The extremization of $\mathcal{L}$ with respect to $P_k(\alpha, \mathbf{r})$ results in an expression for the probability distribution function of PA molecules of type k:

$$P_k(\alpha, \mathbf{r}) = \tag{25}$$

$$q_k(\mathbf{r})^{-1} \exp \left(\sum_{\substack{i=\text{all bead}\\ \text{types}}} \sum_{\substack{j=\text{all bead}\\ \text{types}}} \int\int \mathrm{d}\mathbf{r}'' \mathrm{d}\mathbf{r}' n_i^k(\mathbf{r}'; \alpha, \mathbf{r}) \langle n_j(\mathbf{r}'') \rangle \beta\varepsilon_{ij} g_{ij}(\mathbf{r}', \mathbf{r}'') \right. \tag{26}$$

$$- \sum_{\substack{i=\text{all bead}\\ \text{types}}} \int \mathrm{d}\mathbf{r}' \; \beta\pi(\mathbf{r}') v_i n_i^k(\mathbf{r}'; \alpha, \mathbf{r}) \tag{27}$$

$$- \sum_{\substack{i=\text{ types of}\\ \text{acid-base beads}}} \int n_i{}^k(\mathbf{r}'; \alpha, \mathbf{r}) \left[\ln\left(f_i(\mathbf{r}')\right) + \beta\psi(\mathbf{r}) q_i + \beta\mu^0_{i\ (\text{charged})} \right] \mathrm{d}\mathbf{r}' \tag{28}$$

$$\left. + \sum_{\substack{i=\text{ all bead}\\ \text{types}}} \int \mathrm{d}\mathbf{r}' \frac{(\varepsilon_{PA} - \varepsilon_{sol})\, v_i}{2} \beta [\nabla\psi(\mathbf{r}')]^2 n_i^k(\mathbf{r}'; \alpha, \mathbf{r}) \right) \tag{29}$$

where $q_k(\mathbf{r})$ is the single-chain internal partition function of the PA of type k, which is related to the Lagrange multiplier $\lambda_k(\mathbf{r})$ by $q_k(\mathbf{r}) = exp[1 + \lambda_k(\mathbf{r})]$.

The functional minimization of $\mathcal{L}$ with respect to $\rho_i(\mathbf{r})$ results in:

$$\rho_{sol}(\mathbf{r}) v_{sol} = \exp\left(-\beta\pi(\mathbf{r}) v_{sol}\right) \tag{30}$$

for the solvent,

$$\rho_i(\mathbf{r}) v_{sol} = \exp\left(\beta\mu_i - \beta\psi(\mathbf{r}) q_i - \beta\pi(\mathbf{r}) v_i\right) \tag{31}$$

for the salt anion and cation,

$$\rho_i(\mathbf{r}) v_{sol} = \exp\left(\beta\mu_i - \beta\mu_i^0 - \beta\psi(\mathbf{r}) q_i - \beta\pi(\mathbf{r}) v_i\right) \tag{32}$$

for H^+ and OH^-, and

$$\rho_k(\mathbf{r}) v_{sol} = q_k(\mathbf{r}) \exp\left(\beta\mu_k^*\right) \tag{33}$$

for the PA_k molecule, respectively.

The minimization of $\mathcal{L}$ with respect to $f_i(\mathbf{r})$ results in expression for the chemical equilibria of acid and basic groups (here we assumed $v_{sol} = v_{H^+} = v_{OH^-}$):

$$K^0_{a,\ i} = \exp\left(-\beta\Delta G^0_{a,i}\right) = \frac{f_i(\mathbf{r}) \rho_{\mathrm{H}^+}(\mathbf{r})}{\left(1 - f_i(\mathbf{r})\right) \rho_{sol}(\mathbf{r})} \tag{34}$$

and

$$K^0_{b,\ i} = \exp\left(-\beta\Delta G^0_{b,i}\right) = \frac{f_i(\mathbf{r}) \rho_{\mathrm{OH}^-}(\mathbf{r})}{\left(1 - f_i(\mathbf{r})\right) \rho_{sol}(\mathbf{r})} \tag{35}$$

where $\Delta G^0_{a,i} = \mu^0_{\mathrm{H}^+} + \mu^0_{i,\mathrm{A}^-} - \mu^0_{i,\mathrm{HA}}$ for acid beads (Eq. 34) and $\Delta G^0_{b,i} = \mu^0_{\mathrm{OH}^-} + \mu^0_{i,\mathrm{BH}^+} -$

$\mu_{i,\mathrm{B}}^0$ for basic beads (Eq. 35). The equilibrium constants $K_{a,i}^0$ and $K_{b,i}^0$ are related to the more commonly used equilibrium constants in molar concentrations, k_a and k_b, by $K_a^0 = k_a N_A/(10^{24}\rho_{sol}^{bulk})$ and $K_b^0 = k_b N_A/(10^{24}\rho_{sol}^{bulk})$, where N_A is Avogadro's number and ρ_{sol}^{bulk} is the number density of the solvent in the bulk.

Finally, the extreme of $\mathcal{L}$ with respect to the electrostatic potential, $\psi(\mathbf{r})$, yields:

$$\nabla \cdot (\varepsilon(\mathbf{r})\nabla\psi(\mathbf{r})) = -\langle \rho_Q(\mathbf{r})\rangle \tag{36}$$

This equation is a mean-field version of Poisson's equation for electrostatics (which is a fundamental physical law) because ρ_Q, ψ and ε are ensemble averages rather than values in a single microstate.

Symmetry considerations

A major advantage of MOLT over traditional MD simulations resides in its capability of leveraging the symmetry of the system to lower the computational cost. In this work, we considered three types of ideal self-assembled aggregates: spherical micelles, infinitely long cylindrical fibers and infinite planar lamellas. Each of these ideal morphologies can be described by considering inhomogeneities in a single coordinate, which corresponds to the distance to the center of the micelles, to the central axis of the fibers or to the central plane of the lamellae. This coordinate is named r for the three cases. Therefore, all functions that describe the structure of the system depend only on r, which reduces the cost of numerically solving the molecular theory. These symmetry considerations are applied to the equations of the molecular theory that we derived above for the most general case, which considered inhomogeneities in the three spatial directions. For example, the translational entropy (Eq. 2) becomes:

$$-\frac{S_{translational}}{k_B} = \sum_{\substack{\mathrm{i=\ PA_1,\ldots,PA}_M, \\ \mathrm{anion,cation,H^+,OH^-,sol}}} \int \rho_i(r)\left[\ln\left(\rho_i(r)v_{sol}\right) - 1\right]G(r)dr \tag{37}$$

where $G(r)dr$ is the volume element at a distance r from the center of the micelle, the central axis of the fiber or the central plane of the lamellae. More specifically, $G(r)dr = 4\pi r^2 dr$ for micelles, $2\pi r L dr$ for fibers (where L is the length of the section of fiber under consideration) and $2Adr$ for lamellae (where A is area of the region of lamella).

Special care is required in the definition of the bead number density, Eq. 5,

$$\langle n_i(r)\rangle = \sum_{\mathrm{k=\ PA_1,\ldots,PA}_M} \sum_{\alpha} \int \frac{G(r')}{G(r)} n_i^k(r;\alpha,r')P_k(\alpha,r')\rho_k(r')\,\mathrm{d}r' \tag{38}$$

Note the factor $G(r')/G(r)$, which represents the ratio of volume elements at the position of the center of mass (r') and where the bead number density is being evaluated (r).

The Poisson equation (Eq. 36) takes different explicit forms for the three symmetries under consideration. For planar lamellae, it becomes:

$$\frac{\partial\varepsilon(r)}{\partial r}\frac{\partial\psi(r)}{\partial r} + \varepsilon(r)\frac{\partial^2\psi(r)}{\partial r^2} = -\langle \rho_Q(r)\rangle, \tag{39}$$

for cylindrical fibers:

$$\frac{\partial \varepsilon(r)}{\partial r}\frac{\partial \psi(r)}{\partial r} + \varepsilon(r)\frac{1}{r}\frac{\partial}{\partial r}\left(r\frac{\partial \psi(r)}{\partial r}\right) = -\left\langle \rho_Q(r)\right\rangle \tag{40}$$

and for spherical micelles:

$$\frac{\partial \varepsilon(r)}{\partial r}\frac{\partial \psi(r)}{\partial r} + \varepsilon(r)\frac{1}{r}\frac{\partial^2}{\partial r^2}\left(r\psi(r)\right) = -\left\langle \rho_Q(r)\right\rangle \tag{41}$$

Finally, the boundary conditions for this equation (for the three symmetries under consideration) are:

$$\lim_{r\to\infty} \psi(r) = 0 \tag{42}$$

(choice of zero electrostatic potential in the bulk), and

$$\left.\frac{\partial \psi}{\partial r}(r)\right|_{r=0} = 0 \tag{43}$$

(reflection boundary condition at the origin)

Numerical implementation

The equations resulting from the extremization of MOLT (Eqs. 29-36) and the constrains (Eqs. 19-22) are numerically solved. To do that, we first discretize the r coordinate in layers of thickness $\delta = 0.2$ nm (explicit examples of discretized expressions for the different structural functions are provided in the SIs of Refs.[2] and[3]). The discretization results in a set of non-linear coupled equations, which we solve using a Jacobian-free Newton Method with the KINSOL library.[6,7]

Molecular model

In this section, we discuss the parametrization of MOLT. The volume of solvent molecules was set to $v_{sol} = 0.03$ nm^3, which corresponds to the volume of a water molecule obtained from its density. The volumes of H^+ and OH^- are assumed to be the same as the water molecule. Salt anions and cations have a volume of 0.113 nm^3, which models both the ion and its water of hydration. The salt concentration was set to 0.1 M in all calculations. Note that this parameter is not expected to have a relevant effect on the morphology behavior of the system.[2] The dielectric constants of PA molecules (ε_{PA}) and the aqueous solution (ε_{sol}) were set to 3 ε_0 and 78.4 ε_0, respectively, where ε_0 is the vacuum dielectric permittivity.

Coarse-graining strategy

Our coarse grain (CG) strategy is inspired by the MARTINI force-field for coarse-grain MD simulations.[8] Briefly, a single bead in our theory maps a group of heavy atoms (i.e. non-H atoms). More specifically, we used a single bead in our theory to represent four methylene

units in the alkyl tail, the backbone or the side-chain atoms of the aminoacid (both for lysine and glutamic acid), see examples in Fig. S5. All beads have the same volume (0.113 nm^3).

The acid group of glutamic acid and the basic group of lysine are located in the respective side chains and have pKa = 4.5 (glutamic acid) and pKb = 3.46 (lysine).

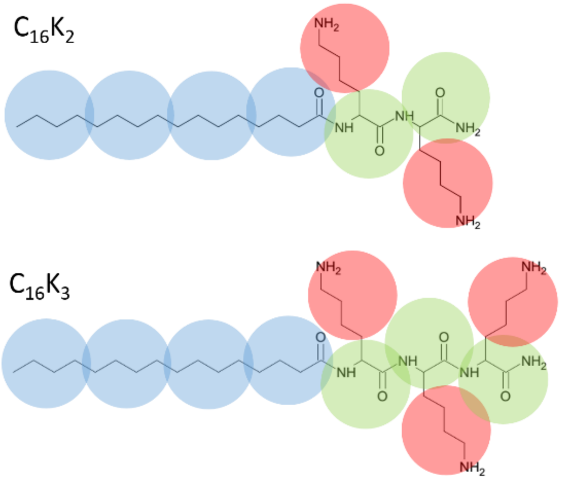

Figure S5: Coarse-graining strategy used for $C_{16}K_2$ and $C_{16}K_3$. Color circles represent the different beads using in MOLT calculations.

Short-range interactions

We model the short-range non-electrostatic effective interactions between two beads in the F_{chem} contribution (Eq. 11) as:

$$g_{ij}(\mathbf{r},\mathbf{r}') = \begin{cases} -\left(\frac{|\mathbf{r}-\mathbf{r}'|}{d}\right)^6 & \text{if } d < |\mathbf{r}-\mathbf{r}'| < d_{cut-off} \\ 0 & \text{otherwise} \end{cases} \tag{44}$$

for all pair of beads i,j. Here, d is the bead diameter (0.47 nm) and $d_{cut-off}$ is a cut-off distance for the potential (we used 2.5δ in this work, which is large enough for all our results to be converged with respect to this parameter).

The interaction parameters $\epsilon_{i,j}$ in the F_{chem} contribution (Eq. 11) account for the strength of the effective interactions between beads i and j. Note that this interaction does not include electrostatic forces, which are accounted for at the level of the mean-field Poisson equation, and repulsions, which are accounted for exactly at the intrachain level (see below) and at a mean-field level for intermolecular repulsions through the use of the packing constraint, Eq. 21. It is also important to mention that the $\epsilon_{i,j}$ are effective interactions parameters (*i.e.*, they describe the difference between the interactions between beads i and j and the interaction of those beads with the solvent). Therefore, $\epsilon_{i,j}$ can take either positive or negative values.[2] Moreover, for this reason, we do not explicitly include interaction parameters for the solvent-solvent and solvent-bead pairs.

In our previous work,[2] we estimated the parameters $\epsilon_{i,j}$ for the alkyl-tail, aminoacid backbone and lysine side-chain beads from the Martini force field. We keep the same interaction parameters in this work (see Figure S6) and assume that the parameters for the glutamic acid side-chain beads are equal to that of the lysine side-chain bead. More details about the estimation of the interaction parameters can be found in the SI of ref.[2]

	tail	backbone	side chain
tail	7,0	-0,93	5,91
backbone	-0,93	-0,93	-0,93
side chain	5,91	-0,93	4,82

Figure S6: $\epsilon_{i,j}$ interaction parameters used in the calculation in units of k_BT

Set of molecular conformations for PAs

MOLT requires as an input a set of molecular conformations for each PA molecule (these sets are then encoded in the functions $n_i^k(\mathbf{r};\alpha,\mathbf{r}')$, see Eq. 5). Each conformation in the set is defined both by the internal dihedral angles of the molecule and its orientation in the space. In principle, a set should contain all possible conformations, but in practice it is enough to use a subset. Following our previous works,[2,3] we used a set of 10^4 conformations for PA and each position of the center of mass, which is large enough to ensure that all calculations are converged with respect to the size of set. The conformations are randomly generated using the Rotational Isomeric State (RIS) model.[9] We only include conformations in which beads do not overlap, thereby the intramolecular steric repulsions are treated exactly by the theory.

Criteria of thermodynamic stability

The proper thermodynamic potential to compare the stability of the aggregates is the semi-grand canonical potential per molecule in excess to the bulk, which, for a mixture of two PAs, is defined as:

$$\omega\left(T,x_1,\{\mu_i\}\right)=\frac{\Omega^*(T,V,n_1,n_2,\{\mu_i\})-\Omega^*(T,V,n_1=0,n_2=0,\{\mu_i\})}{n_1+n_2} \tag{45}$$

where V is the volume, T is the temperature, $\{\mu_i\}$ represents the chemical potentials of all ions, and n_i is the number of PA molecules of type i. Note that ω is defined as the difference between the semi-grand canonical potential, Ω^* (given by Eq. 18), for the system with PAs and that for a bulk system (system without PAs) having the same V, T and μ_i.

In the case where the self-assembled aggregate completely fits in V, and the concentrations of free PAs in the solution surrounding the aggregate are very small, then ω becomes independent of V because increasing the volume of the calculation box only adds a bulk region (whose free energy is then substracted in the calculation of ω) . The normalization by n_1+n_2 makes ω an intensive quantity. Thus, ω allows us to compare the relative stability of systems with the same T and $x_1=n_1/(n_1+n_2)$, but different V (volume of the calculation

box), $n_T = n_1 + n_2$, and morphology (spherical micelle, cylindrical fiber or planar lamella). In the main text, we refer to ω as the "free energy per molecule" for simplicity.

Theoretical analysis of the morphology coexistence in PA mixtures

To use MOLT to predict the equilibrium morphology for PA mixtures, we solve the theory for different ideal morphologies: spherical micelles, infinitely long cylindrical nanofibers and planar lamellae. These calculations require to fix the experimental conditions of the solution (pH and ionic strength) and the molecular structure and molar fractions of PA_1 and PA_2 in the mixture. For each morphology, we first scan the total aggregation number, $n_T = n_1+n_2$, for a fixed composition $x_1 = n_1/(n_1 + n_2)$, and then find the one that minimizes the free energy per molecule, ω.[2,3] We repeat this procedure for different values of x_1 and plot the equilibrium ω v*s* x_1 for each different morphology, see a typical plot in the scheme in Figure S7 (same as Figure 1 in the main text).

In the example in Fig. S7, the curve that describe the free energy per amphiphile of the most stable morphology *vs* x_1 has a concave-up region, then the free energy of the system in that region can be lowered by having a coexistence of two different morphologies (*i.e.*, micelles, M and fibers, F). To analyze this coexistence region, let us denote as n_i^j ($i = 1, 2$ and $j = M, F$) to the total number of molecules of PA i in the aggregate of type j and $n_T^j = n_1^j+n_2^j$ to the total number of PAs in the aggregate of type j . In the coexistence region, the composition of the assemblies M and F is fixed to x_1^M and x_1^F, respectively, and their free energies per amphiphile are $\omega(x_1^M)$ and $\omega(x_1^F)$, respectively. Since any global composition x_1 within the coexistence region must be achievable by combining these two assemblies, we require $x_1^M < x_1 < x_1^F$. The free energy per amphiphile of the coexisting-micelle system therefore is

$$\omega^c(x_1) = \omega(x_1^M)\frac{n_T^M}{n_T} + \omega(x_1^F)\frac{n_T^F}{n_T} \tag{46}$$

Note that the ratio n_T^j/n_T is the fraction of all PA molecules in assemblies of type j. We sum and subtract $\omega(x_1^F)$ at the RHS, use $n_T = n_T^M + n_T^F$ and regroup

$$\omega^c(x_1) = \left[\omega(x_1^M) - \omega(x_1^F)\right]\frac{n_T^M}{n_T} + \omega(x_1^F) \tag{47}$$

We now use the following identity:

$$\frac{x_1 - x_1^F}{x_1^M - x_1^F} = \frac{n_T^M}{n_T^F} \tag{48}$$

to finally obtain:

$$\omega^c(x_1) = \left[\omega(x_1^M) - \omega(x_1^F)\right]\frac{x_1 - x_1^F}{x_1^M - x_1^F} + \omega(x_1^F) \tag{49}$$

This is the equation of a line connecting points $x_1^M, \omega(x_1^M)$ and $x_1^F, \omega(x_1^F)$.

Now, we focus on the question on how to identify the points x_1^M and x_1^F. The line $\omega^c(x_1)$ that minimizes the energy of the coexisting system is an extremum with respect to the choice

of these points

$$\left.\frac{\partial \omega^c(x_1)}{\partial x_1^M}\right|_{x_1, x_1^F} = \left.\frac{\partial \omega^c(x_1)}{\partial x_1^F}\right|_{x_1, x_1^M} = 0 \tag{50}$$

Calculating these derivatives (from Eq. 46) results in the following expressions:

$$\frac{\partial \omega^c(x_1^M)}{\partial x_1} = \frac{\partial \omega^c(x_1^F)}{\partial x_1} = \frac{\omega(x_1^M) - \omega(x_1^F)}{x_1^M - x_1^F} \tag{51}$$

These equations show that the points x_1^M and x_1^F should be selected so that the line $\omega^c(x_1)$ is tangent to the curve $\omega(x_1)$ at those points.

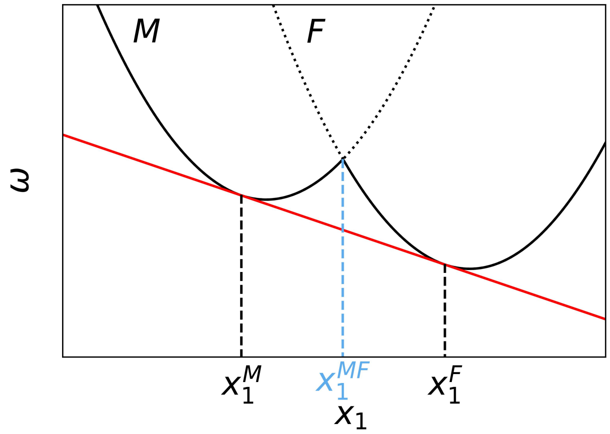

Figure S7: (Same as Figure 1 in the main text) Scheme of the free energy per PA (ω) as a function of the molar fraction of PA_1 in the mixture, x_1 for the spherical micelle (M) and cylindrical nanofiber (F) morphologies. The solid red line is tangent to the M and F curves at points x_1^M and x_1^F, respectively. For all global compositions between between x_1^M and x_1^F, there is a coexistence between micelles with composition x_1^M and fibers with composition x_1^F.

Additional AFM data

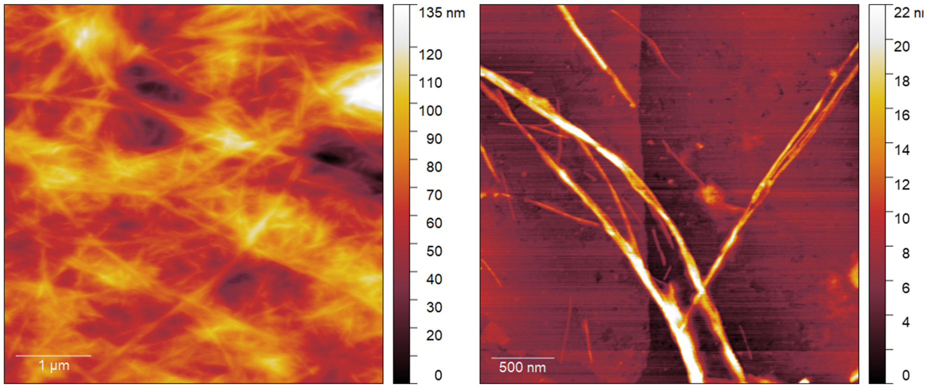

Figure S8: AFM images for C_{16}KK/C_{16}EE mixture for pH and a molar fraction of C_{16}KK $x_1 = 0.8$.

SAXS measurements

The SAXS data were analyzed using three different models: core-shell sphere, core-shell cylinder, and core-shell parallelepiped. The mathematical expressions for each model are provided below, following the definitions used in the SasView documentation (https://www.sasview.org/docs/user/user.html). In all cases, a gaussian size distribution was considered for the cores and the scattering length densities (SLDs) were fixed as follows: $sld_{\text{core}} = 7.8 \cdot 10^{-6}$ Å^{-2} (aliphatic chains), $sld_{\text{shell}} = 12.5 \cdot 10^{-6}$ Å^{-2} (peptide heads), and $sld_{\text{solvent}} = 9.46 \cdot 10^{-6}$ Å^{-2} (water).

Core-Shell sphere model

The scattering intensity for a core-shell sphere is given by:

$$I(q) = \frac{\text{scale}}{V} F^2(q) + \text{background} \tag{52}$$

where the form factor amplitude $F(q)$ is defined as:

$$F(q) = \frac{3}{V}\left[V_c(\rho_c - \rho_s)\frac{\sin(qr_c) - qr_c\cos(qr_c)}{(qr_c)^3} + V(\rho_s - \rho_{\text{solv}})\frac{\sin(qr) - qr\cos(qr)}{(qr)^3}\right] \tag{53}$$

and V_c and V are the volumes of the core and the whole particle, respectively. ρ_c, ρ_s, and ρ_{solv} are the scattering length densities of the core, shell, and solvent. r_c and r are the core and total (core + shell) radii.

Core-shell cylinder model

For a cylindrical core-shell structure, the scattering intensity is given by:

$$I(q,\alpha) = \frac{\text{scale}}{V} F^2(q,\alpha)\sin(\alpha) + \text{background} \tag{54}$$

where:

$$\begin{aligned} F(q,\alpha) = (\rho_c - \rho_s) V_c & \frac{\sin\left(q\frac{L}{2}\cos\alpha\right)}{q\frac{L}{2}\cos\alpha} \frac{2J_1\left(qr_c\sin\alpha\right)}{qr_c\sin\alpha} \\ + (\rho_s - \rho_{\text{solv}}) V & \frac{\sin\left(q\left(\frac{L}{2}+T\right)\cos\alpha\right)}{q\left(\frac{L}{2}+T\right)\cos\alpha} \frac{2J_1\left(q(r_c+T)\sin\alpha\right)}{q(r_c+T)\sin\alpha} \end{aligned} \tag{55}$$

α is the angle between the cylinder axis and the scattering vector q, $J_1(x)$ is the first-order Bessel function. V_c and V are the core and total (core + shell) volumes, respectively. r_c is the core radius and T is the shell thickness. L is the core cylinder length. ρ_c, ρ_s, and ρ_{solv} are the scattering length densities of the core, shell, and solvent.

Core-Shell Parallelepiped Model

The core-shell parallelepiped model describes a rectangular solid with a core-shell structure. The core is defined by axis, A (thickness), B (width) and C and one scattering length density (SLD) (longitude). The shell is defined by "rims", with different thickness and SLD, on each pair of faces. The model does not include a shell on the edges where the faces meet. The scattering intensity is given by:

$$I(q) = \frac{\text{scale}}{V} \int_0^{\frac{\pi}{2}} \int_0^{\frac{\pi}{2}} |F(q,\alpha,\beta)|^2 \sin\alpha \, d\alpha \, d\beta + \text{background} \tag{56}$$

where α is the the angle between the long axis of the parallelepiped (C-axis) and the scattering vector q, β is the rotation angle around the long axis (C-axis) and the integral averages over all orientations of the parallelepiped.

The scattering amplitude $F(q,\alpha,\beta)$ is:

$$\begin{aligned} F(q,\alpha,\beta) = (\rho_{\text{core}} - \rho_{\text{solvent}}) & S(Q_A,A)S(Q_B,B)S(Q_C,C) \\ & + (\rho_A - \rho_{\text{solvent}})\left[S(Q_A,A+2t_A) - S(Q_A,A)\right] S(Q_B,B)S(Q_C,C) \\ & + (\rho_B - \rho_{\text{solvent}})S(Q_A,A)\left[S(Q_B,B+2t_B) - S(Q_B,B)\right] S(Q_C,C) \\ & + (\rho_C - \rho_{\text{solvent}})S(Q_A,A)S(Q_B,B)\left[S(Q_C,C+2t_C) - S(Q_C,C)\right] \end{aligned} \tag{57}$$

where A, B and C are the lengths of the parallelepiped along the x, y and z-axis respectively. t_A, t_B and t_C are the shell thickness on the A, B and C-faces. ρ_{core} and ρ_{solvent} are the core and solvent scattering length density. ρ_A, ρ_B and ρ_C are the SLD of the shell on the A, B and C-faces. V is the total volume of the parallelepiped including the shell.The components

of the scattering vector Q along the different axes are:

$$Q_A = q \sin\alpha \sin\beta \tag{58}$$
$$Q_B = q \sin\alpha \cos\beta \tag{59}$$
$$Q_C = q \cos\alpha \tag{60}$$

For the present case, t_B and t_C were set to 0, and the only core–shell feature was modeled along the thickness of the parallelepiped (A-Axis + t_A). Since the SAXS curves of $C_{16}KK/C_{16}EE$ mixtures at $x_1 = 0.6$ and 0.8 exhibit a broad peak in the high-q region - partially masked by the bump associated with the parallelepiped form factor- the data were fitted using a combination of a core-shell parallelepiped model (to represent the lamellae) and a Gaussian peak. The Gaussian component accounts for the correlations between stacked lamellar units forming short-range domains.

The Gaussian peak model in SasView represents a symmetric peak centered at a position q_0, and is described by:

$$I(q) = A \exp\left(-\frac{(q-q_0)^2}{2\sigma^2}\right) \tag{61}$$

where A is the amplitude, q_0 is the peak center (related to inter-domain spacing), and σ determines the peak width (inversely related to the domain size). This model provides a simple yet effective way to capture broad structural features without assuming specific geometric shapes.

Additional SAXS data

Figures S9 and S10 show additional SAXS curves and their corresponding fits, which were not included in the main text.

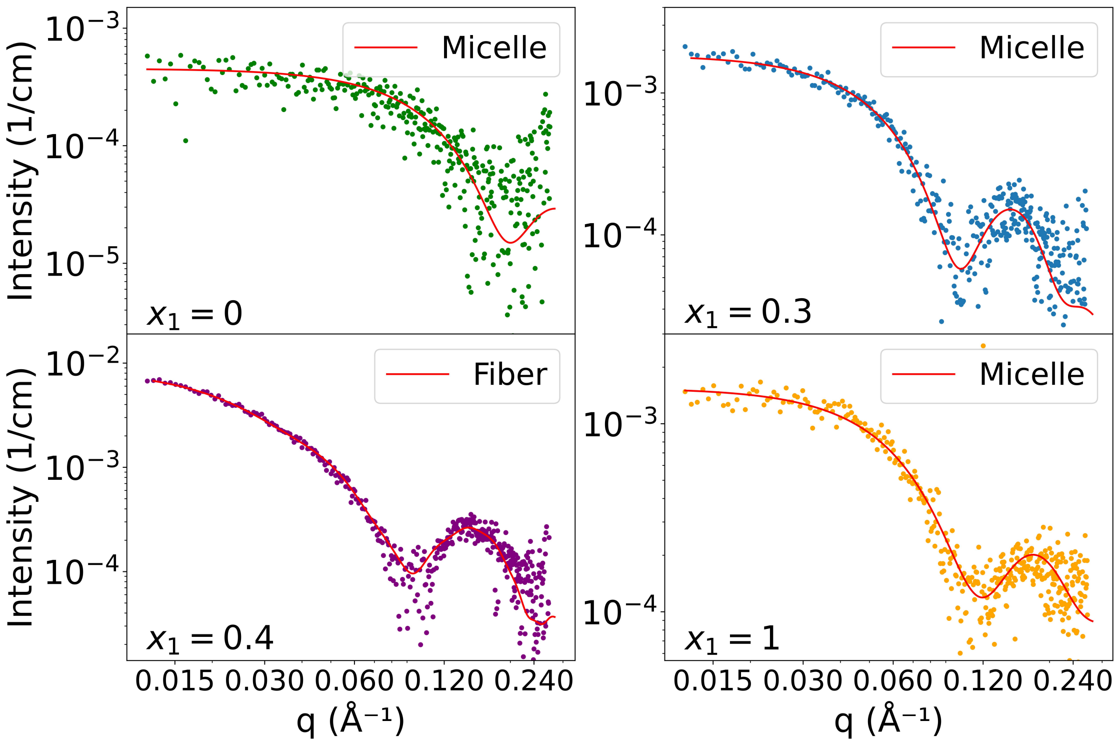

Figure S9: SAXS curves for the mixture $C_{16}K_3/C_{16}E_3$ at pH 8.

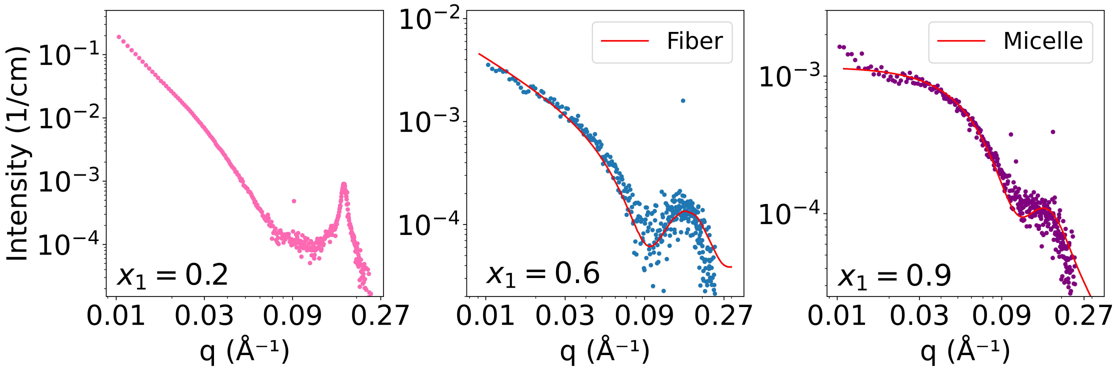

Figure S10: SAXS curves for the mixture $C_{16}K_2/C_{16}E_2$ at pH 6.

Figure S10 shows SAXS curves for $C_{16}K_2/C_{16}E_2$ mixtures at pH 6 for three compositions. The curve at $x_1 = 0.2$ displays a low-q slope close to -2 and a strong peak at intermediate q, features that are typically associated with stacked lamellar structures. Unfortunately, the presence of this peak precludes a good fitting with the lamella + Gaussian peak model used for pH 9. In contrast, the SAXS curves at $x_1 = 0.6$ and $x_1 = 0.9$ are well described by fiber and micelle models, respectively. These results are consistent with MOLT predictions and confirm the expected morphological behavior of the system at pH 6.

References

(1) Szleifer, I.; Carignano, M. Tethered polymer layers. *Advances in chemical physics* **1996**, *94*, 165–260.

(2) Zaldivar, G.; Vemulapalli, S.; Udumula, V.; Conda-Sheridan, M.; Tagliazucchi, M. Self-Assembled Nanostructures of Peptide Amphiphiles: Charge Regulation by Size Regulation. *The Journal of Physical Chemistry C* **2019**, *123*, 17606–17615.

(3) Zaldivar, G.; Conda-Sheridan, M.; Tagliazucchi, M. Molecular basis for the morphological transitions of surfactant wormlike micelles triggered by encapsulated nonpolar molecules. *Langmuir* **2021**, *37*, 3093–3103.

(4) Zaldivar, G.; Perez Sirkin, Y. A.; Debais, G.; Fiora, M.; Missoni, L. L.; Gonzalez Solveyra, E.; Tagliazucchi, M. Molecular theory: A tool for predicting the outcome of self-assembly of polymers, nanoparticles, amphiphiles, and other soft materials. *ACS omega* **2022**, *7*, 38109–38121.

(5) Israelachvili, J. N.; Mitchell, D. J.; Ninham, B. W. Theory of self-assembly of hydrocarbon amphiphiles into micelles and bilayers. *Journal of the Chemical Society, Faraday Transactions 2: Molecular and Chemical Physics* **1976**, *72*, 1525–1568.

(6) Gardner, D. J.; Reynolds, D. R.; Woodward, C. S.; Balos, C. J. Enabling new flexibility in the SUNDIALS suite of nonlinear and differential/algebraic equation solvers. *ACM Transactions on Mathematical Software (TOMS)* **2022**,

(7) Hindmarsh, A. C.; Brown, P. N.; Grant, K. E.; Lee, S. L.; Serban, R.; Shumaker, D. E.; Woodward, C. S. SUNDIALS: Suite of nonlinear and differential/algebraic equation solvers. *ACM Transactions on Mathematical Software (TOMS)* **2005**, *31*, 363–396.

(8) Marrink, S. J.; Risselada, H. J.; Yefimov, S.; Tieleman, D. P.; De Vries, A. H. The MARTINI force field: coarse grained model for biomolecular simulations. *The journal of physical chemistry B* **2007**, *111*, 7812–7824.

(9) Rubinstein, M.; Colby, R. H. *Polymer physics*; Oxford university press, 2003.
